# Functional Gradient Metallic Biomaterials: Techniques, Current Scenery, and Future Prospects in the Biomedical Field

**DOI:** 10.3389/fbioe.2020.616845

**Published:** 2021-01-18

**Authors:** Hongyuan Shi, Peng Zhou, Jie Li, Chaozong Liu, Liqiang Wang

**Affiliations:** ^1^School of Aeronautical Materials Engineering, Xi'an Aeronautical Polytechnic Institute, Xi'an, China; ^2^Institute of Orthopaedic & Musculoskeletal Science, University College London, Royal National Orthopaedic Hospital, London, United Kingdom; ^3^State Key Laboratory of Metal Matrix Composites, Shanghai Jiao Tong University, Shanghai, China

**Keywords:** implants, biomedicine, functional gradient material, additive manufacturing, graded structures

## Abstract

Functional gradient materials (FGMs), as a modern group of materials, can provide multiple functions and are able to well mimic the hierarchical and gradient structure of natural systems. Because biomedical implants usually substitute the bone tissues and bone is an organic, natural FGM material, it seems quite reasonable to use the FGM concept in these applications. These FGMs have numerous advantages, including the ability to tailor the desired mechanical and biological response by producing various gradations, such as composition, porosity, and size; mitigating some limitations, such as stress-shielding effects; improving osseointegration; and enhancing electrochemical behavior and wear resistance. Although these are beneficial aspects, there is still a notable lack of comprehensive guidelines and standards. This paper aims to comprehensively review the current scenery of FGM metallic materials in the biomedical field, specifically its dental and orthopedic applications. It also introduces various processing methods, especially additive manufacturing methods that have a substantial impact on FGM production, mentioning its prospects and how FGMs can change the direction of both industry and biomedicine. Any improvement in FGM knowledge and technology can lead to big steps toward its industrialization and most notably for much better implant designs with more biocompatibility and similarity to natural tissues that enhance the quality of life for human beings.

## Introduction

Today's medicine desperately needs modern materials and methods that have multiple applications meeting different goals. In this regard, the biomedical field deals with technology development that helps to enhance the quality of human life; hence, each parameter is of crucial importance. The success of biomedical devices mostly depends on the materials used to make them; a variety of materials, including metals and their alloys, ceramics, composites (Zhu et al., 2018; Wang L. et al., 2019), and polymers, are used in the biomedical field. Moreover, implant design is the other significant factor; currently, there are many types of implant designs, and most of them are in an attempt to mimic the function of natural organs. Generally, each device consists of only one component with a unified structure. However, medical devices should meet some requirements, such as biocompatibility, osseointegration, strength, corrosion, and abrasion resistance, low elastic modulus, fatigue durability, and chemical similarity with biological tissues, and the traditional designs may not satisfy these varied requirements (Ehtemam-Haghighi et al., 2016; Okulov et al., 2017; Wang S. et al., 2019). For instance, some devices can cause premature failure or failure after long-term use in the human body (Niinomi and Nakai, 2011; Liu et al., 2016); these issues stem from the fact that one or more basic mechanical or biological requirements are not fully met. Bone structure is always in a remodeling procedure, which makes it possible to react with its environment and stressors in this regard. According to Wolff's law, bone tissue is produced and strengthens in the direction of mechanical stress lines. Wolff's law clinically has been proven by the apparent fabrication of osteophytes around an arthritic joint and also by the occurrence of osteoporosis due to the stress-shielding effect (Burke and Goodman, 2008). The stress-shielding effect is the bone density reduction (osteopenia) that happens as a result of stress removal from the bone by an implant, specifically orthopedic implants, and it is a major problem leading to failure of the implant and increasing the cost of surgery (Zhang B. et al., 2018). Taking this information into account, the necessity to find modern solutions is urgent and leads to the development of functional graded materials (FGMs). This FGM concept is a promising method to control the stress-shielding effect. For example, both radial and axial FGM dental implants considerably reduced the stress-shielding effect in the periphery of bone tissue (Asgharzadeh Shirazi et al., 2017), and FGM utilization can also prevent this phenomena in femoral prostheses (Oshkour et al., 2013) and other biomedical implants. In this regard, Hedia and Fouda (2013) show that FGM material with vertical gradation of hydroxyapatite (HA) at the end of the stem tibia to collagen at the upper layers of the tibia plate can reduce the stress-shielding effect by 78%. Moreover, FGM materials can also withstand high sliding and contact forces (Suresh, 2001) and have better and stronger adhesion, shear bond strength, and fatigue properties (Matsuo et al., 2001; Henriques et al., 2012).

The FGM concept was first introduced during the 1980s by Japanese scholars, whose main goal was the reduction of thermal stresses in metallic-composite materials utilized in reusable rocket engines (Koizumi and Niino, 1995). Nowadays, there are lots of studies devoted to FGM design (Mohd Ali et al., 2020). Over time, the benefits of this concept were realized in the modern biomedical and tissue engineering field because one of the characteristics of living tissues and natural structures is their functional gradation that can be seen in bone (Wegst et al., 2015), the wings of various insects (Appel et al., 2015), fish armor (Zimmermann et al., 2013), gecko skin (Arzt, 2006; Jagnandan et al., 2014), etc. (Liu et al., 2017).

**Figure 1:**
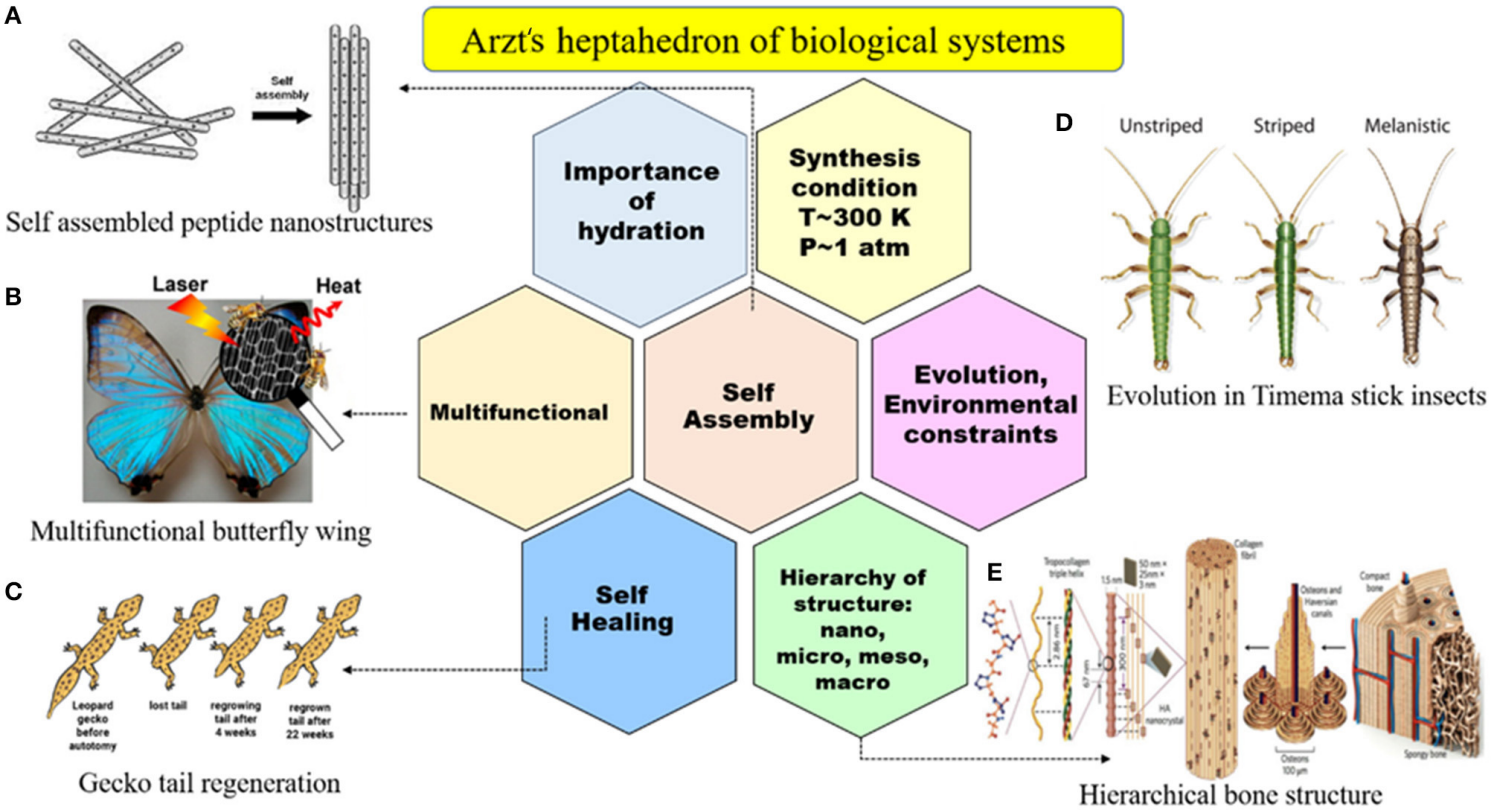
Arzt heptahedron indicating the seven key features of biological systems in nature that synthetic biomaterials still cannot reach, including self-assembly, multifunctionality, self-healing, the hierarchy of structure, evolution against environmental constraint, synthesis under T~300 K and P~1 atm, and hydration. Natural examples: **(A)** self-assembled peptide nanostructures with permission from Gazit (2007), **(B)** multifunctional butterfly wing with permission from Miyako et al. (2013), **(C)** gecko tail regeneration with permission from Gecko Tail Regeneration (2011), **(D)** evolution in Timema stick insects with permission from Nosil et al. (2018), and **(E)** hierarchical bone structure with permission from Wegst et al. (2015).

The main objective of tissue engineering is to fabricate biological substitutes that well mimic the structure and properties of the live organ and structure in order to treat the injured regions of the body (Vacanti, 2006). Nature is full of various structures known as spatially heterogeneous composites with customizable properties. Biological systems have seven key characteristics, known as the Arzt heptahedron, based on which all bioinspired conceptions must be designed, and it is shown in Figure 1. These seven key characteristics of biological systems are (1) self-assembly, (2) multifunctionality, (3) self-healing, (4) hierarchy of structure, (5) evolution against environmental constraint, (6) synthesis under T~300 K and P~1 atm conditions, and (7) hydration necessity. FGM biomaterials try to obey these criteria to fully mimic the natural structures because these natural components have excellent proficiency, and manmade parts still have a long way to go to reach them. There are lots of successful biomimetic FGM designs with promising properties that show the ability of utilizing this concept in biomedical implants (Chavara et al., 2009; Bin Qasim et al., 2020). One of the commercially used examples in this field is superelastic nickel titanium (Ni-Ti) orthodontic arch wires with graded functionality (Braz Fernandes et al., 2019).

**Figure 2:**
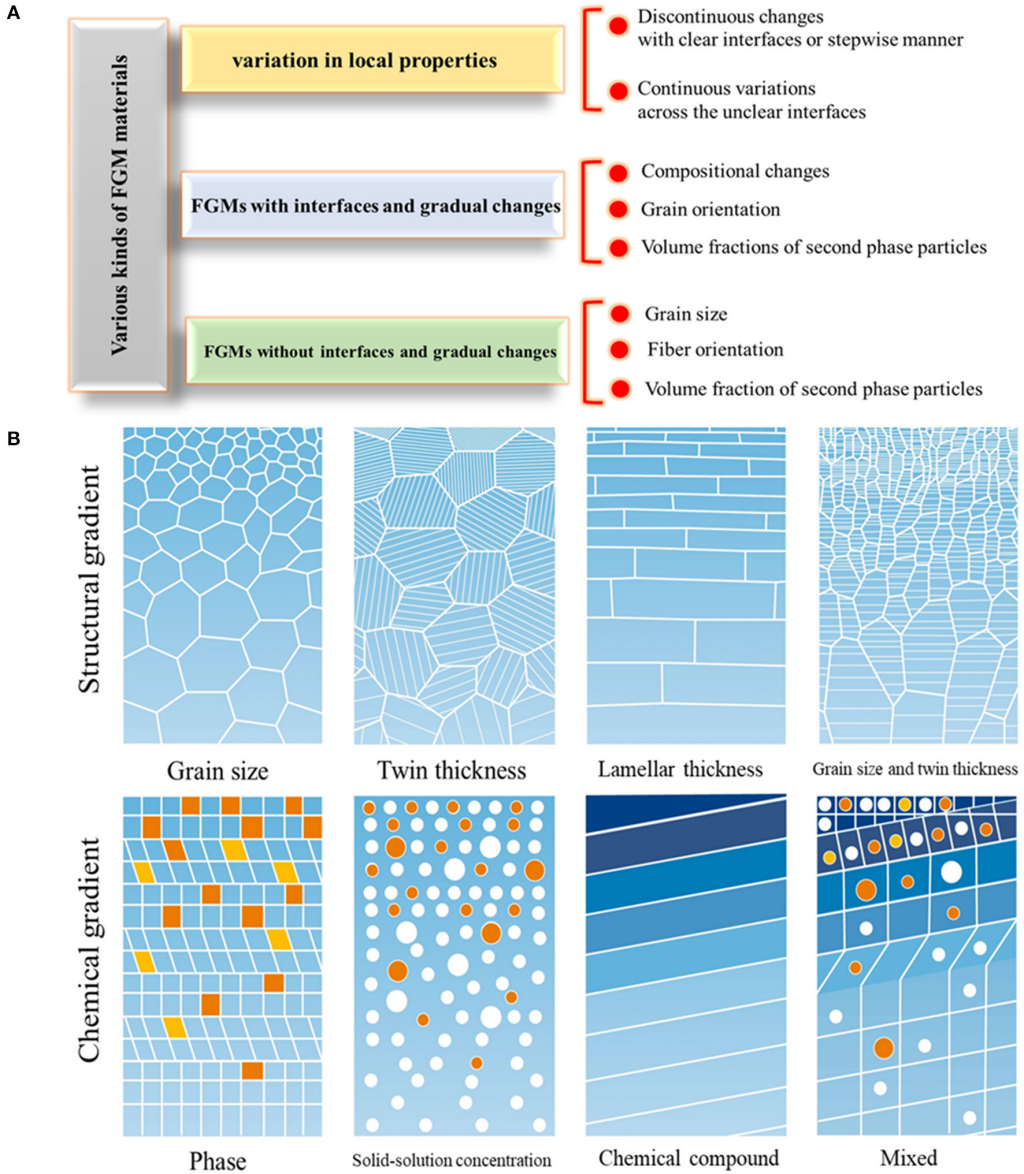
The various kinds of FGM materials. **(A)** The categorization, **(B)** schematic representing the various kinds of structural and chemical gradients in FGMs with permission from Li X. et al. (2020).

In this regard, FGM design in tissue engineering is considered an innovative scheme for improving biomedical device performance and aims to reach specified multifunctional characteristics through spatial, structural, or compositional gradation, leading to tailored properties (Kawasaki and Watanabe, 1997). The most important advantage of FGMs is their capability in providing tailored morphological features that lead to the occurrence of graded physical and mechanical properties in a specific direction. FGMs have gradual transitions from composition, constituents, microstructure, grain size, texture, porosity, etc., along with one or more directions that lead to functional property changes. Also, according to interface condition, FGMs are classified into continuous and discontinues types. Figure 2 schematically represents the FGM structure and types. Moreover, to produce biomimetic FGM parts, it is better to use seven common biological structure parts found in nature, such as fibers, helical, layered, tubular, gradient, cellular, suture, and overlapped components (Liu et al., 2017).

Metallic implant materials, including titanium (Ti) and its alloys, stainless steel, Co-Cr alloys, and NiTi shape memory alloys, are utilized commonly in cases in which high mechanical properties are required. Among these metallic materials, Ti attracted much attention because of its supreme biocompatibility, excellent corrosion resistance, low density, good mechanical properties, etc. (Rack and Qazi, 2006; Gode et al., 2015; Wang et al., 2017a; Attarilar et al., 2019a,b, 2020). For the case of Ti utilized in dental and orthopedic surgeries, the main goal of functionalization is to improve the osseointegration of the implant by surface engineering (Yang et al., 2006; Wang et al., 2015; Zhu et al., 2016; Ding et al., 2019; Wang Q. et al., 2020). Nowadays, additive manufacturing is also used in order to mitigate the stress-shielding effect by the production of Ti structures with gradation in pore size and shape from the surface to the center of the part (Yuan et al., 2019). Metallic FGMs have lots of applications as biomaterials utilized in various parts of the body. Finite element analysis by Enab (2012) indicates that the FGM tibia tray has superb biomechanical performance, mitigating stress-shielding and shear stress issues. It is shown that downward gradual elastic modulus variation of the tibia tray from 40 to 110 GPa in the vertical direction reduces the stresses. Also, the metallic FGMs have numerous applications in orthopedic surgery (Sola et al., 2016) and dentistry (Senan and Madfa, 2017). Metallic implants are very suitable to maintain the requirements for bone implant application, load-bearing parts, and scaffolds, and this review paper especially focuses on the materials utilized in dental and bone applications. Due to the growing number of older populations in the world, the need for *de novo* multifunctional implants grows rapidly day by day; hence, this subject is of crucial importance and may open new horizons in the aspect of metallic FGMs.

## FGM Manufacturing Methods

**Figure 3:**
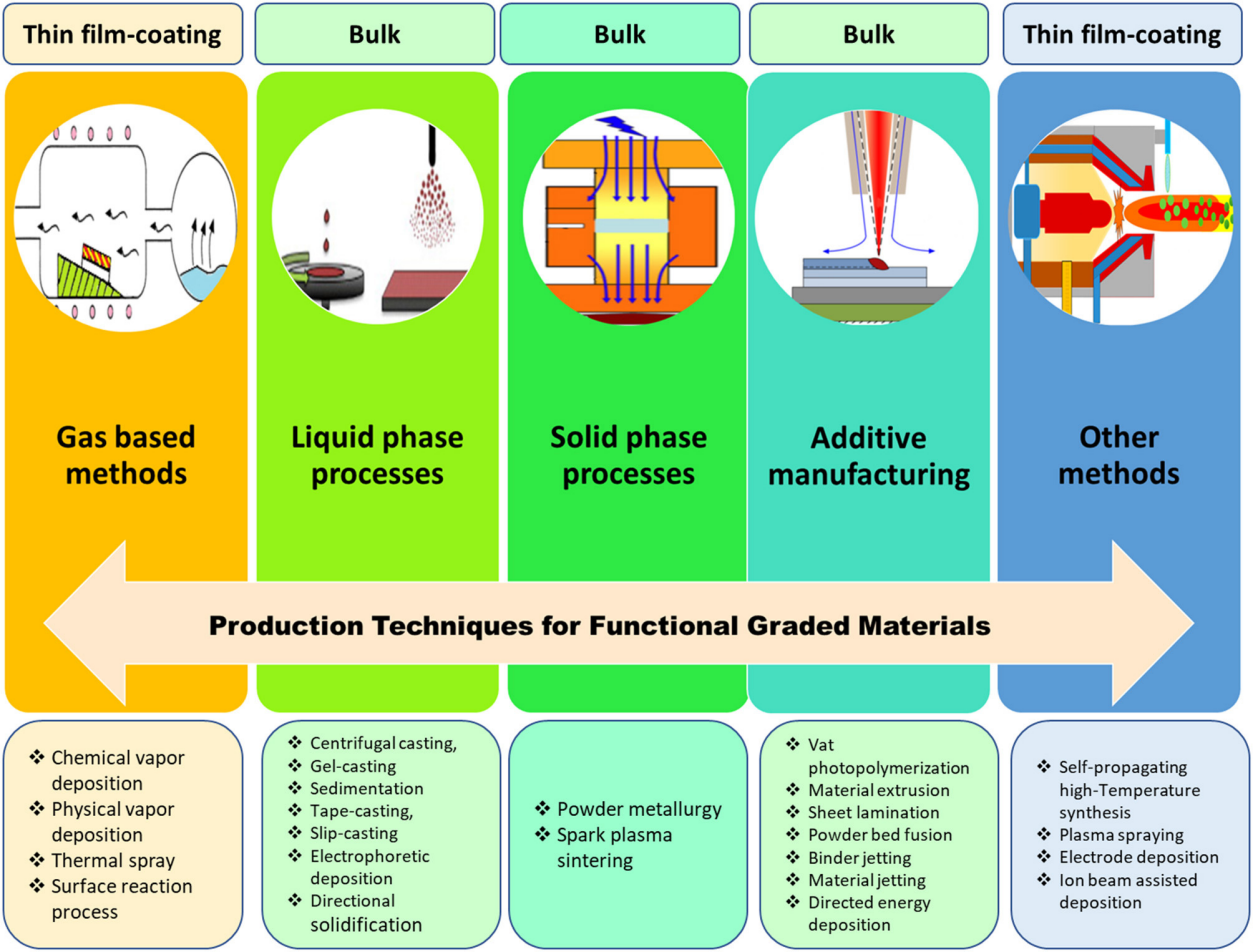
Various technologies for the production of FGMs resulting in bulk samples, coating, or thin-film fabrication: gas-based technique, such as chemical vapor deposition (CVD) (Choy, 2003); liquid-phase process (Faure et al., 2013); solid-phase process, such as spark-plasma-sintering (SPS) (Xie et al., 2011); additive manufacturing, such as laser engineered net shaping (LENS) (Cong and Ning, 2017); and other methods, such as plasma-spraying process (Unabia et al., 2018).

The manufacturing technique is of key importance to attain high-quality FGMs with the desired gradation and properties; therefore, numerous methods have been proposed and used so far; each of which has its own pros and cons, and these methods are listed in Figure 3. The FGM production techniques can be categorized into four main processes: gas-based methods, liquid phase (Chen et al., 2000), solid phase (Tripathy et al., 2018), and additive manufacturing processes. Also, there are some other methods, such as ion beam–assisted deposition. Recently, additive manufacturing (AM) techniques have become a very popular method for the production of FGMs because these methods have the capability to manufacture complex porous structures with even nanometric resolution (Zhang et al., 2019). These AM techniques are among the best options for biomedical and implant applications because they are fast and economic, and most importantly, they can be precisely adjusted to meet patient-specific needs, such as shape, dimension, and even texture of the related live tissues (Javaid and Haleem, 2018; Culmone et al., 2019).

### Gas-Based Techniques

#### Chemical Vapor Deposition

**Figure 4:**
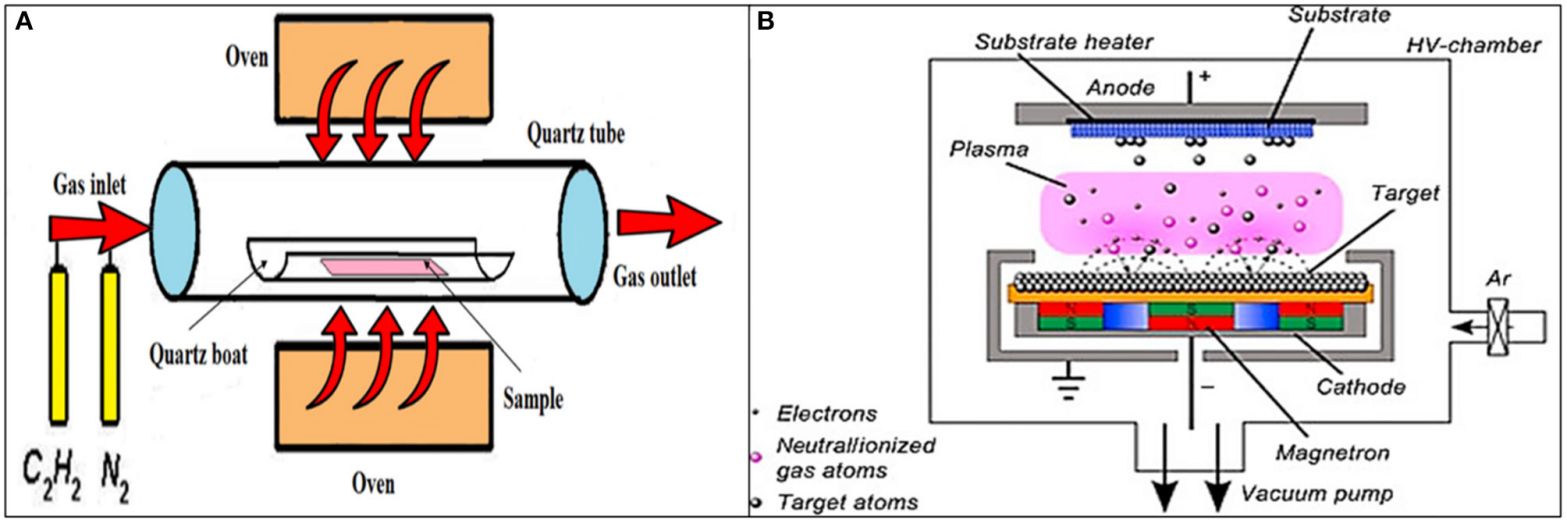
**(A)** The schematic representation of the chemical vapor deposition (CVD) method, including its various parts like an oven, quartz boat and tube, gas inlet, and outlet sections. **(B)** The schematic representation of the physical vapor deposition (PVD) method with permission from Mishra et al. (2019).

One of the most common ways to produce FGMs is to produce surface coatings and induce surface gradation. Gas-based processes are among these coating-based methods, and chemical vapor deposition (CVD) is a popular method in this group. In the CVD method, various energy sources can be used, including light, heat, and plasma, to deposit materials on a surface. The used gases are usually in the form of hydride, bromide, and chloride. The gradation of deposited material can be tailored by temperature, gas ratio, gas type, flow rate, etc. (Hirai, 1995). The beneficial aspects of the CVD method in FGM fabrication are the potential to control the continuous variation of the composition, its low-temperature condition, and the resultant near-net designated shape of samples (Naebe and Shirvanimoghaddam, 2016). In the CVD technique, a carbon source in the gas phase and a kind of energy source (light, plasma, or a resistively heated coil) is utilized to transfer energy to a gaseous carbon molecule. In this process, hydrocarbons, including methane, carbon monoxide, and acetylene, etc., are used as carbon sources. These hydrocarbons flow in a quartz tube while heating in an oven (~720°C); a schematic of the CVD technique can be found in Figure 4A. Due to energy application, the hydrocarbon chains are broken, and this leads to the production of pure carbon molecules; hence, the carbon can diffuse toward the heated substrate that is coated with a catalyst species (usually transition metals, such as Ni, Fe, or Co), where it binds. The CVD process has several advantages, such as low power input, lower temperature range, relatively high purity, and most importantly, the possibility to scale up the process (Endo et al., 1993). Liu et al. (2006) show the effectiveness of the plasma-enhanced CVD process in the development of an anticorrosion diamond-like carbon (DLC) FGM coating deposition on a Nitinol (NiTi) substrate. The produced DLC coating has a 150-nm-thick graded layer with excellent adhesivity to the substrate and effective corrosion protection in simulated body fluids.

#### Physical Vapor Deposition

The gas-based physical vapor deposition (PVD) technique is among the well-known approaches to produce various thin films and coatings. In this process, the material transforms from the condensed phase to a vapor phase and subsequently rearranges to the condensed phase as a thin film or coating on the substrate. Figure 4B shows a schematic of the PVD device. The PVD method has numerous applications, including optics, electronic, chemical, semiconductors, solar panels, food packaging, TiN coatings in cutting tools, etc. The advantages of the PVD process are its ability to produce various kinds of coatings (organic and inorganic), environmentally friendliness, and achieving durable coatings with favorable properties, but unfortunately, it is operating under high temperatures and vacuum conditions. The magnetron sputtering PVD systems can be used to produce ion-substituted Ca-P-based coatings on the surface of an implant, and these coatings, such as hydroxyapatite (HA) show significant influence on cell interactions, including cell proliferation, adhesion, and differentiation (Qadir et al., 2019). The PVD method is a proper candidate to develop a reproducible preparation of nano rough titanium thin films with biological properties (Lüdecke et al., 2013). Also, a new kind of gradient DLC coating is produced by the plasma source ion PVD system, and it can have some applications on artificial mechanical heart valves (Yin et al., 1999).

The plasma spray technique, as one of the PVD methods, can be used to produce three-layered-FGM hydroxyapatite (HA)/Ti-6Al-4V coatings with progressive variation of microhardness, Young's modulus, microstructure, and porosity between layers (Khor et al., 2003). The excellent tensile adhesion strength of these coatings, fracture toughness, microhardness, etc., of this FGM makes it a suitable choice for biomedical applications. The beneficial properties of compositionally graded doped (HA)/Ti-6Al-4V FGM produced by the plasma spray technique are also confirmed by Ke et al. (2019). Moreover, the *in vitro* human experiments by osteoblast cell culture and tests against *E. coli* and *S. aureus* bacterial species prove its superb biological performance. This FGM shows favorable interfacial mechanical and antibacterial properties (due to an MgO and Ag_2_O mixture with HA) for possible use in load-bearing orthopedic and dental implants (Ke et al., 2019).

### Liquid Phase Processes

#### Electrophoretic Deposition

**Figure 5:**
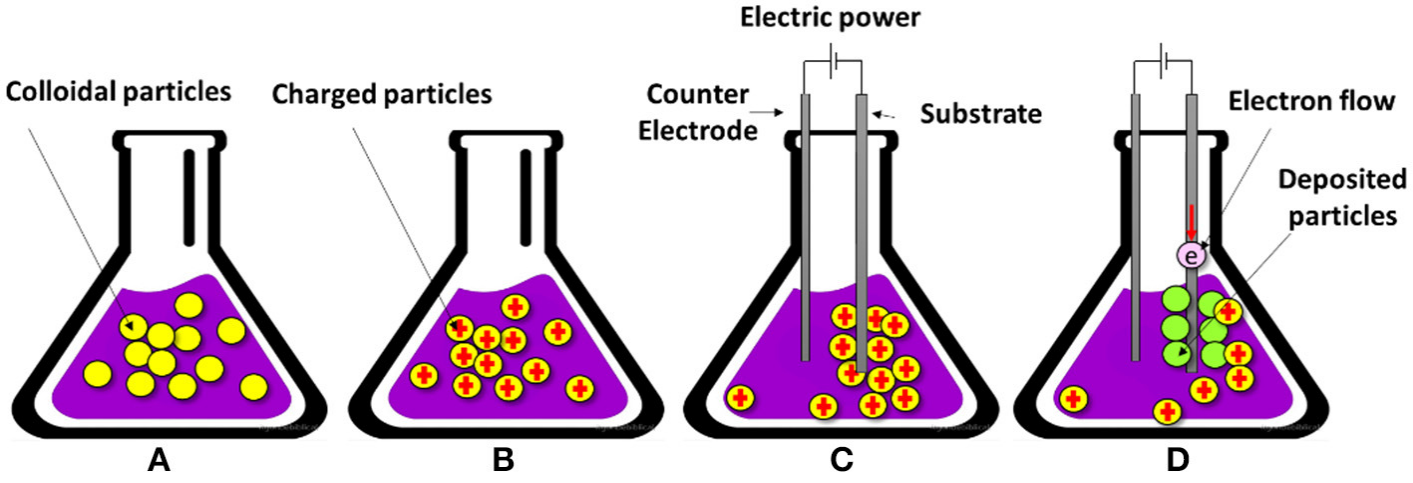
The schematic of four stages of the electrophoretic deposition (EPD) technique: **(A)** colloidal particle dispersion, **(B)** charged particles due to electrochemical interactions, **(C)** electrophoresis, and **(D)** deposition of a firm layer of particles on the substrate.

In the electrophoretic deposition (EPD) technique, a stable colloidal suspension is used in which, due to the existence of an electric field, the charged particles are moved and deposited on a conductive substrate with the oppositely charged condition. As observed in Figure 5A, the colloidal particles are randomly dispersed and are able to move freely in the solvent suspension. In Figure 5B, the surface of the particles are charged due to electrochemical equilibrium, and in Figure 5C, the external electrical field causes the preferential movement of charged particles toward the oppositely charged electrode, which is the substrate. Finally, in Figure 5D, the adsorbed charged particles get some electrons and transform into the firmly deposited layer of particles on the surface of the substrate (Amrollahi et al., 2016). EPD has the potential to fabricate a variety of materials from traditional to advanced materials from nanometric thin films and coatings to a thick film, and from porous scaffold parts to highly compact coatings and FGMs. In EPD, both AC and DC electrical fields can be used, but DC is more common (Amrollahi et al., 2016). This method has lots of advantages with versatile application: some of its pros include simple device and utilization, short processing time, economic, facile modification, desirable dense packing of particles, high-quality microstructure, fabrication of geometrically complicated shapes, and simple control of the thickness and morphology (Sarkar et al., 2012). Sun et al. (2009) utilize a cathodic EPD method in order to fabricate multilayered HA-chitosan FGM coatings with HA particles, and this method has the potential to produce layers with different thicknesses ranging between 2 to 200 μm. The resultant composite chitosan-heparin layers can be used for surface modification of HA-chitosan coatings and improving blood compatibility.

### Solid Phase Processes

#### Powder Metallurgy

Powder metallurgy (PM) is one of the well-known solid-phase processes to produce FGMs in which a graded powder material is mixed with a specific proportion stacked together in a continuous manner or *via* step-by-step stages. Then, the stacked material is compacted by pressing to achieve a dense condition, and the resultant compact part is sintered in a specified range of temperature to reach a 100% dense part (Madan and Bhowmick, 2020). The most significant stages of the PM process, respectively, are powder weighing, powder mixing, compaction, and sintering. Compaction is usually performed under a controlled atmosphere with low temperatures, and sintering should be done at a high temperature range. To improve the quality of the PM part, it is common to do some postprocessing, such as coining, repressing, and resintering. PM methods are widely used in the production of FGM parts, specifically ceramic FGMs. One of the important advantages of these methods is their ability to produce intricate and complicated shapes out of any metallic or ceramic powders, and it is the best method to produce FGMs out of solid constituents (Tripathy et al., 2017). Shahrjerdi et al. (2011) produce metal-ceramic composite FGM by a pressur-less sintering method using pure Ti and HA; the compositional gradation was from the metallic (Ti) end to the ceramic (HA) end. In order to optimize biocompatibility and mechanical properties, Watari et al. (2004) fabricate a Ti/HA FGM by PM method, and this specimen shows the better maturation of freshly formed bone cells in the HA-rich region than the Ti-rich zone. The produced graded structure causes proper osteogenesis and mechanical and stress relaxation properties. Overall, these studies indicate that FGM fabrication and the resultant gradation affect the tissue reaction in a graded manner; hence, it is possible to tailor the biological response of tissues by the development of FGM biomaterials.

#### Spark Plasma Sintering

**Figure 6:**
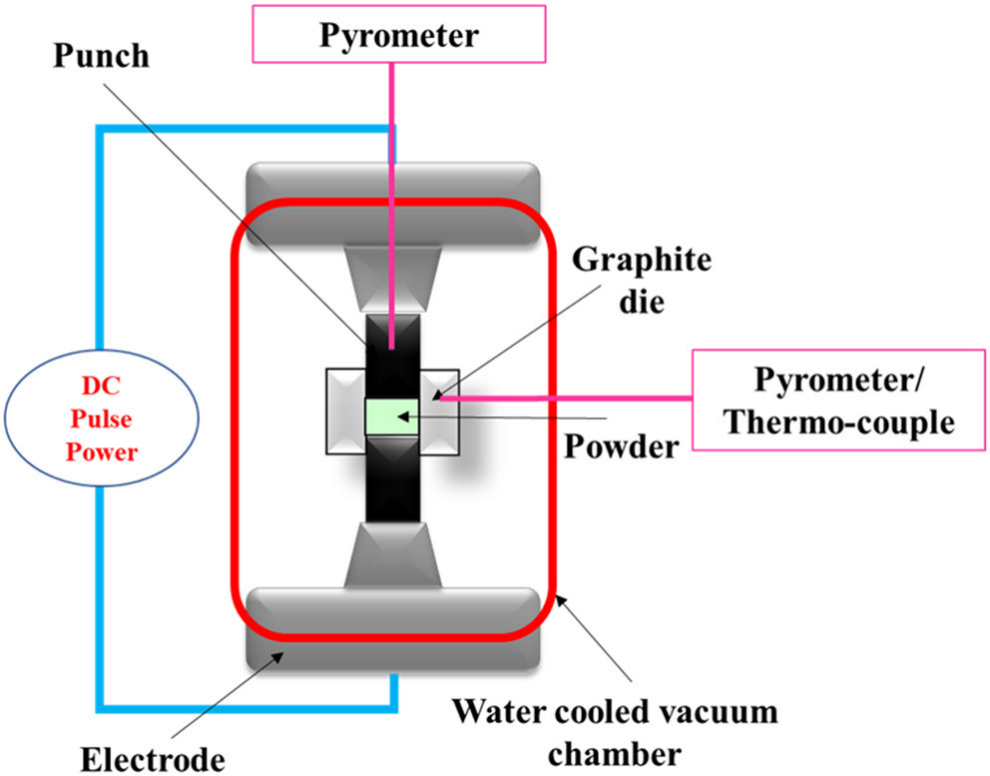
The schematic of spark plasma sintering.

The spark plasma sintering (SPS) method is a compressive, solid-state method in which a pulsed electric current energizing sintering is used (Tokita, 2010) and can be efficiently utilized to produce FGM parts. Kondo et al. (2004) use the SPS method to fabricate titanium nitride/apatite functionally graded implants with acceptable mechanical properties and new bone formation around the femur of a rat model. In the SPS method, electric current is utilized in the densification step, in which a pulsed DC current is directly transferred to a graphite die and the powder compact; this process is schematically shown in Figure 6. In the SPS method, the compact part is heated internally; hence, high heating rates (~1,000 K/min) are possible, and also, it leads to a very fast sintering process (a few minutes). The characteristics of this method include short holding times, fast heating, fast cooling, and the potential to achieve completely dense parts at relatively low temperatures. Using the SPS method in the fabrication of HAp/zirconia composites with biomedical applications helps to inhibit undesirable chemical reactions that lead to reduction in the biocompatibility and mechanical properties of the part. The SPS process with a proper temperature range and high pressing loads ensures the proper characteristics of HA ceramic composites without the accuracy of decomposition reactions; hence, the resultant HAp/zirconia composites are five to seven times stronger and seven times tougher with the suitable biological response (Shen et al., 2001).

### Additive Manufacturing Methods

**Figure 7:**
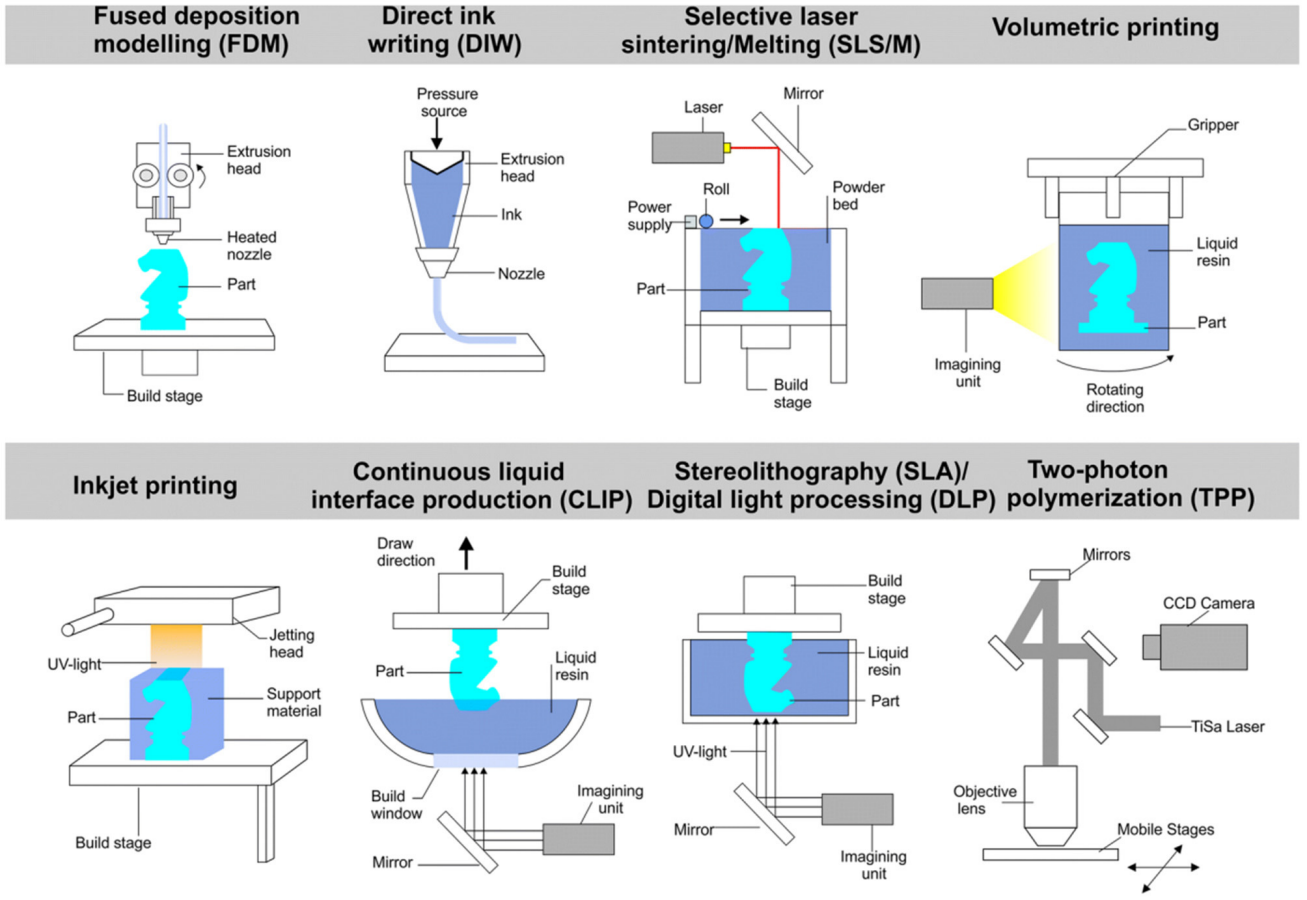
Schematic representation of some additive manufacturing methods with permission from Yan et al. (2020).

Additive manufacturing (AM) technologies, also known as 3-D printing methods, are among the most recent FGM fabrication procedures (Zhang B. et al., 2016), and some of them are shown in Figure 7. They have the potential to fabricate very complex and intricate porous parts with high resolution. They are simple and direct methods that do not need any dies, tooling, joining, sintering, or assembling steps. AM technology has numerous advantages and is a unique procedure to produce different structural and industrial parts. In particular, it has a big impact on the biomedical field. Some of its advantages that can be mentioned are its economic nature, ability to mass produce, potential to produce very complex parts, repeatability, shorter time to market, ability to use various materials (organic or inorganic), etc. (Attaran, 2017). One of the significant benefits of 3-D printing technology is the possibility to use a computer-aided design (CAD) technique that enables fabricating completely patient-specific implants (Jardini et al., 2014; Mobbs et al., 2017). During AM processing, first, the 3-D CAD model is converted to a printable digital files (such as.STL files), and then the processed data are grouped as thin, 2-D slices using slicing software. Subsequently, the developed slices are fed to the 3-D printer device to build the final parts in a layer-by-layer manner (Zhang L. et al., 2016). There are numerous AM methods, each of which has its benefits and limitations. The most important methods in the fabrication of metallic FGM structures are selective laser melting (SLM) and electron beam melting (EBM) methods, and more information about the AM methods can be found in (Bikas et al., 2016; Awad et al., 2018; Ngo et al., 2018). AM manufacturing techniques have a sizeable impact on the biomedical field. Table 1 summarizes some of the studies that have been done on the biomedical application of AM-manufactured FGMs.

**Table 1:** The application and the biological and mechanical properties of mostly AM-fabricated metallic FGMs.

**Method**	**Material**	**Mechanical properties**	**Biological properties**	**Application**	**References**
SLM	Ti6Al4V	Young's modulus in the range of cortical bone. The highest strength and toughness in honeycomb structures with supporting structure in the outer layer.	–	Orthopedic	Xiong et al., 2020
SLM	Ti6Al4V	Mechanical properties in the range of cortical bone. Small pores with ~900 μm in core regions increase mechanical strength.	Large pores, about 1,100 μm in the outer surface, enhances cell penetration and proliferation.	Load-bearing implants	Onal et al., 2018
SLM	Ti6Al4V	The variation in unit cell orientation affects the mechanical properties; this change is a function of the geometrical dimension of the unit cell size. There is a functional relationship between elastic modulus and compressive strength.	–	Bone implant	Weißmann et al., 2016
SLM	Ti6Al4V	AM-produced porous FGM can decrease the elastic modulus up to 80% and enhance the biomechanical performance.	–	Bone scaffolds and orthopedic implants	Wang et al., 2017b
SLM	Ti6Al4V	Porosity variation strategy (diamond lattice structures) results in an elastic modulus of 3.7–5.7 GPa and yield strength of 27.1–84.7 MPa, which lie in the range of the corresponding mechanical properties of cancellous bone and cortical bone.	–	Bone scaffolds	Zhang X.-Y. et al., 2018
SLM	Pure Ti	Diamond porous FGM scaffold production with a good geometric reproduction, possessing a wide range of graded volume fraction. The elastic modulus is comparable to cancellous bone and can be tailored by tuning the graded volume fraction.	–	Bone implant	Han et al., 2018a
SLM	CoCr	Pillar-octahedral-shape CoCr cellular structures with a porosity range of 41–67% indicate stiffness, strength, and energy absorption values that are similar to natural bone.	–	Metallic orthopedic implants	Limmahakhun et al., 2017
SLM	CoCrMo	FGM design (square pore cellular structures) decrease the stiffness and weight up to 48% compared to the traditional fully dense stem.	–	Femoral stem implant	Hazlehurst et al., 2014a
SLM	CoCrMo	FGM structure (square pore) reduced the stress-shielding effect without compromising the bone strength. The most effective design is the full porous stems with an axially graded stiffness.	–	Hip implant	Hazlehurst et al., 2014b
EBM	Ti6Al4V	The deformation response of graded meshes is the weighted percentage of stress-strain response of each uniform mesh constituent. By tailoring the relative density and volume fraction, the graded meshes can achieve high strength and energy absorption values.	–	Implants that can withstand abrupt impact fractures.	Li et al., 2016
EBM	Ti6Al4V	–	AM manufactured interconnected gradient porous architecture enhances cellular functions, including adhesion, proliferation, mineralization, and synthesis of actin and vinculin proteins.	Medication of segmental bone defects and bone remodeling	Nune et al., 2016
EBM	Ti6Al4V	Aimed to inhibit the stress-shielding effect by decreasing the elastic modulus mismatch between the bone tissue and titanium alloy implant.	3-D printed interconnected porous FGM is conducive to osteoblast cell functions, including proliferation, adhesion, calcium deposition, and synthesis of proteins, such as actin, vinculin, and fibronectin.	Bone implants	Nune et al., 2017
EBM	Ti6Al4V	The weighted average gradient porosities of 65–21% show high compressive strength and hardness and suitable elastic modulus for bone implant application.	–	Treatment of segmental bone defect	Surmeneva et al., 2017
EBM	Ti6Al4V	Regular diamond lattice indicates suitable compression strength and elastic modulus to implant application. Uniaxial compression behavior along the direction of gradation can be well-predicted by a simple rule of mixtures approach.	–	Orthopedic implant applications	van Grunsven et al., 2014
EBM	Ti6Al4V	Periodic cellular structures with a 49%−70% porosity range with mechanical properties (effective stiffness, compressive strength values) suitable for loading conditions.	–	Hip and mandible implants	Parthasarathy et al., 2011
EBM	Ti6Al4V	The porous FGM (open-cell cubic structure) has a compressive strength with a transition region between 4 and 8 mm and is superior to a sharp interface.	–	Biomedical implants	Wu et al., 2018
SLM	316L stainless steel	High density of subgrain boundaries and dislocations is responsible for good plasticity, and the considerable number of voids induces premature instability and fracture.	Higher biocompatibility and good biological performance.	Medical implant applications.	Kong et al., 2018
Laser metal deposition	Stainless steel, HS6-5-2	Low porosity and no delamination occurrence were seen. According to microhardness tests, gradient materials sintered in the N_2_-10% H_2_ atmosphere and reinforced with the VC carbide have the maximum hardness.	–	Possible biomedical application.	Matula and Dobrzański, 2006
Laser cladding	Dissimilar stainless steel (SS)-zirconium (Zr)	Functionally graded deposition in dissimilar materials resulted in production of disintegrated structure and numerous longitudinal and horizontal cracks. Possibly micro-cracks are the result of large thermal stress build-up during the layer-by-layer AM process.	–	–	Khodabakhshi et al., 2019

#### Selective Laser Melting (SLM) and Electron Beam Melting (EBM)

The SLM method, also known as direct metal laser melting (DMLM) or laser powder bed fusion (LPBF), is among the most famous rapid prototyping techniques, and it uses a high power-density laser to melt and fuse metallic powders together. The SLM process has several successive steps from digital data preparation to the ejection of the produced part from the building platform. At the first step, stereolithography (.STL) files are utilized to generate the slice data for each layer, and then the CAD data are transferred to the SLM machine. Initially, the first thin layer of metal powder lies on a tray, and then a laser beam with a high energy-density beam melts and fuses the preferred regions of the powder layer according to the CAD data. After that, the building platform is lowered, and the next layer of powder is deposited on the previous layer. Then, the laser beam begins to scan a new layer. This cycle is repeated numerous times until the 3-D part is completely produced. Finally, the completed 3-D part can be removed from the platform manually or by special devices, and also, the loose powder is removed from the surface of the part (Yap et al., 2015). The SLM process has numerous benefits that make it one of the most used 3-D printing methods, including the short time to market-, no restriction in geometry and ability to produce very complex and porous parts, relatively low cost, no need for assembly steps, etc. (Yap et al., 2015).

The SLM technique is able to produce complex porous FGM scaffolds; for example, the gradient porosity variation strategy is suitable for orthopedic implants to mimic the natural bone structure. Xiong et al. (2020) studied the production of porous Ti6Al4V FGM parts for orthopedic applications by utilization of the SLM method. The gradient porous cellular structures have two kinds of unit cells (honeycomb and diamond-like unit cells). The porosity of samples was in the range of 52–67% with the approximate pore size between 420 and 630 μm, and then the mechanical and physical properties as well as their deformation behavior was studied. The resultant Young's modulus was comparable with the cortical bone (Xiong et al., 2020). In another study, Onal et al. (2018) use the SLM technique to produce Ti6Al4V porous scaffolds with three strut diameters (0.4, 0.6, and 0.8 mm), two gradations (dense-in, dense-out), and BCC structure. The obtained mechanical properties of all designed scaffolds fall in the cortical bone range. Also, the results indicate that dense-in scaffolds with small pores located in the core region and large pores on the outer surface are the best condition for load-bearing implants (Onal et al., 2018).

The EBM method is one of the famous layer-by-layer techniques. It has great potential in the fabrication of high-resolution metallic components (Chern et al., 2020; Tan et al., 2020) and the near net shape parts with intricate geometries (Wang et al., 2016). The process begins with the selective melting of discrete powder layers *via* an electron-beam gun under the vacuum condition, and this melting stage is accomplished by the energy emission *via* the electron beam of a tungsten filament, which can be effectively controlled by two magnetic coils (Galarraga et al., 2016). In the EBM process, each slice is separated into two prescribed zones, including contours and squares. Initially, the contour zone known as an interface between the sample and the surrounding powders is 3-D printed. After that, the square zone acting as the inner zone between these boundary and contour zones is 3-D printed by EBM. One of the advantages of EBM is the use of a vacuum chamber that restrains any impurity and contamination accumulation and leads to the fabrication of high-quality specimens possessing good mechanical properties (Wang et al., 2018; Wang P. et al., 2020).

**Figure 8:**
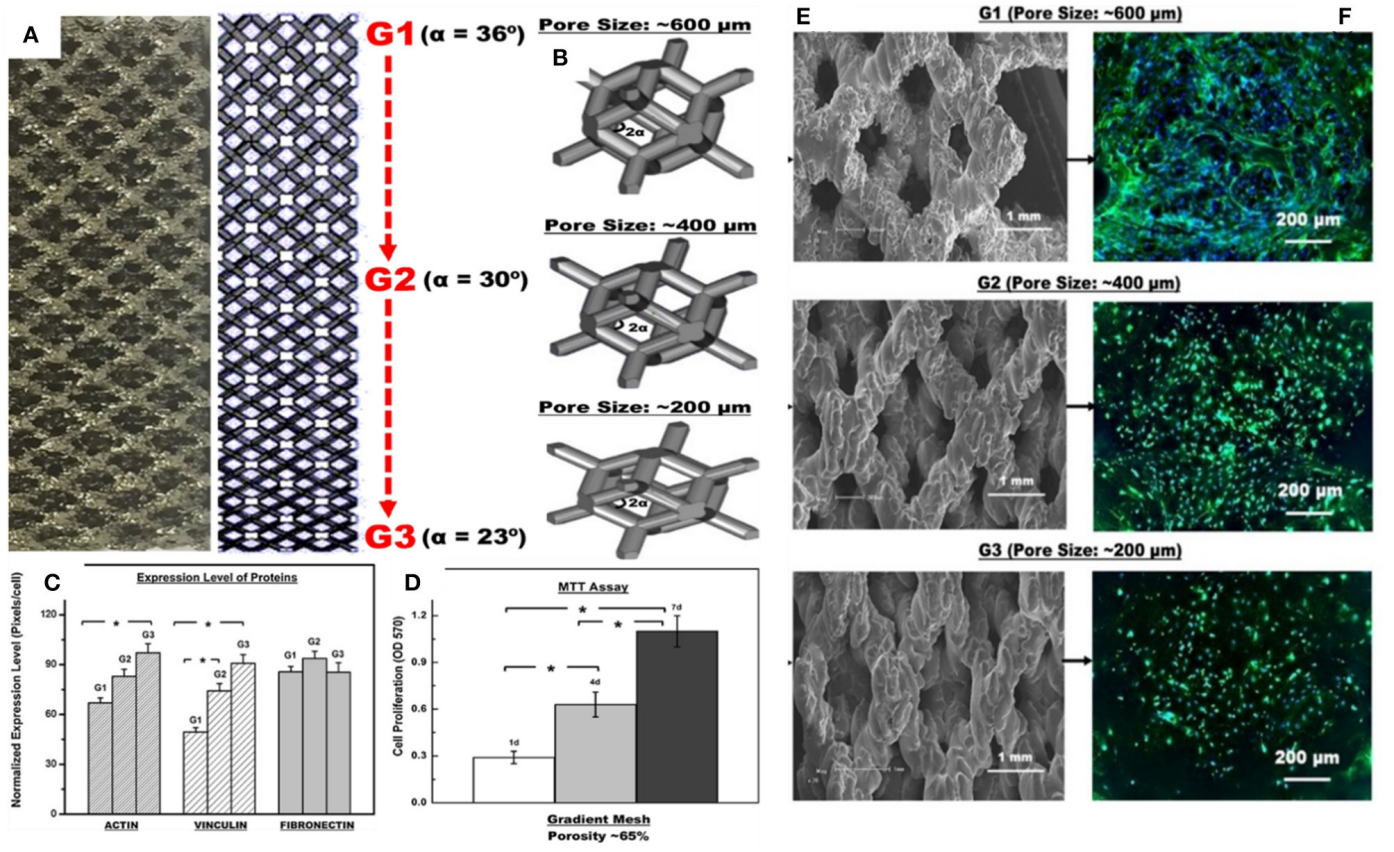
Biological response of osteoblasts to Ti-6Al-4V FGM mesh arrays with permission from Nune et al. (2017): **(A)** micrograph of gradient mesh structure with 2-D CAD design of a gradient mesh structure with unit cells ranging from G1 to G3, **(B)** CAD models of G1 to G3 unit cells with 36, 30, and 23 angles of α with respective pore sizes of ~600, 400, and 200 μm, **(C)** normalized expression level of proteins (actin, vinculin, and fibronectin) histogram, **(D)** cell proliferation histogram, **(E)** scanning electron micrographs of gradient cellular structure, and **(F)** high magnification fluorescence micrographs illustrating the distribution of extracellular fibronectin (green), DAPI stained nucleus (blue) on pre-osteoblasts seeded on FGMs with pore size ranging from ~600 to ~200 μm after 14 days of culture. *The significant difference with 95% confidence level (*p* < 0.05).

A multiple-layered, gradient cellular Ti6Al4V scaffold was produced by the EBM technique, and the mechanical properties were studied by uniaxial compression testing (Surmeneva et al., 2017). Five types of structures with various designs (two layers, three-layered structures, BCC and diamond-like structures with different unit cell sizes) and with weighted average gradient porosities of 65–21% resulted in compressive strength and Young's modulus in the range of 31–212 MPa and 0.9–3.6 GPa, respectively. Also, the results indicate that the lattice cell design significantly affects the failure mechanism. Nune et al. (2017) investigate the biological response of osteoblasts to Ti-6Al-4V FGM mesh arrays fabricated by the EBM method. The gradient structure is composed of different unit cells from G1 to G3 (rhombic dodecahedrons) because it is reported that these types of unit cells have more production flexibility (Li et al., 2014). They are shown in Figure 8A. The CAD models of unit cells ranging from G1 to G3 with 36, 30, and 23 α angles with respective pore sizes of about 600, 400, and 200 μm are shown in Figure 8B. Also, the scanning electron micrographs (SEM) of these gradient mesh structures are, respectively, shown in Figure 8E. It is worth mentioning that the strut thickness was fixed at ~500 μm. Figure 8C shows a histogram of the normalized expression level of proteins (actin, vinculin, and fibronectin). There is not any significant difference in the normalized expression level of fibronectin protein on the struts of the FGM with respect to different regions, but overall, the cellular structure is conducive to the synthesis of proteins. In addition, the cell proliferation histogram in Figure 8D shows that the proliferation of osteoblast cells repeatedly increased with time such that the proliferation rate was about 47%/day on the seventh day. The G1 to G3 regions of FGM demonstrated substantial variations in the distribution of cell nuclei (Figure 8F), and it declined from G1 to G3 with higher density on G1. This reduction in the distribution of nuclei can be related to the topography of the strut surface. In general, this EBM-produced FGM improved the osteoblasts response, including protein synthesis, cell adhesion, proliferation, and calcium deposition (Nune et al., 2017).

## The Functional Design of FGMs in Biomedical Applications

Metallic implants are usually designed to serve as load-bearing implants and are not aimed at being temporary used. They usually have a permanent nature except for some magnesium-based ones for specific applications in which the corrosion rate is of significant importance. Although they have excellent mechanical strength and uniaxial tensile and compression strength, the metallic implants have one major limitation because of their Young's modulus, which is much larger than natural human bone and leads to stress shielding issues and early failure of the implant. The cortical bone has a Young's modulus value between 5 and 23 GPa, and these values are, respectively, about 114, 190, 45, 44, and 120 for Ti-6Al-4V, 316L stainless steel, pure Mg, WE43 Mg alloy, and pure Ti (Haghshenas, 2017). Because of this big difference, the FGM design in metallic implants aim to solve this issue. Satisfactorily good mechanical gradation can be achieved by FGM design and controlling the related variants. Also, by smart hierarchical surface design and fabrication of biomechanical and chemical bonding in surface layers of FGMs, the bone cells can attach and differentiate easily on them and facilitate the treatment procedure (Liu et al., 2017; Bahraminasab and Edwards, 2019; Bai et al., 2019). This kind of gradation is also seen in natural systems and can lead to variations in the mechanical properties of the part; one of the good examples is the bone shown in Figure 1E that can be used in load-bearing applications (Wegst et al., 2015). There are lots of examples in nature, including fish scales and shark teeth, where their unique structure can resist contact and impact forces (Chen et al., 2012). This concept can be used in metallic parts in order to enhance mechanical strength against contact deformation and damage (Suresh, 2001), cracking (Bao and Wang, 1995), and improvement of other mechanical properties (Islam et al., 2020). Sedighi et al. (2017) produce a five-layered Ti/HA composite FGM for dental implant applications. In this study, Ti and HA powders were mixed with different Ti-to-HA ratios (100, 90:10, 80:20, 70:30, and 60:40), and then samples were sintered by the SPS method, the results confirm the graded microhardness values and microstructure differences. Also, some other researchers studied the effect of these gradual changes using AM methods (Lima et al., 2017; Han et al., 2018b).

### FGM Dental Implants

**Figure 9:**
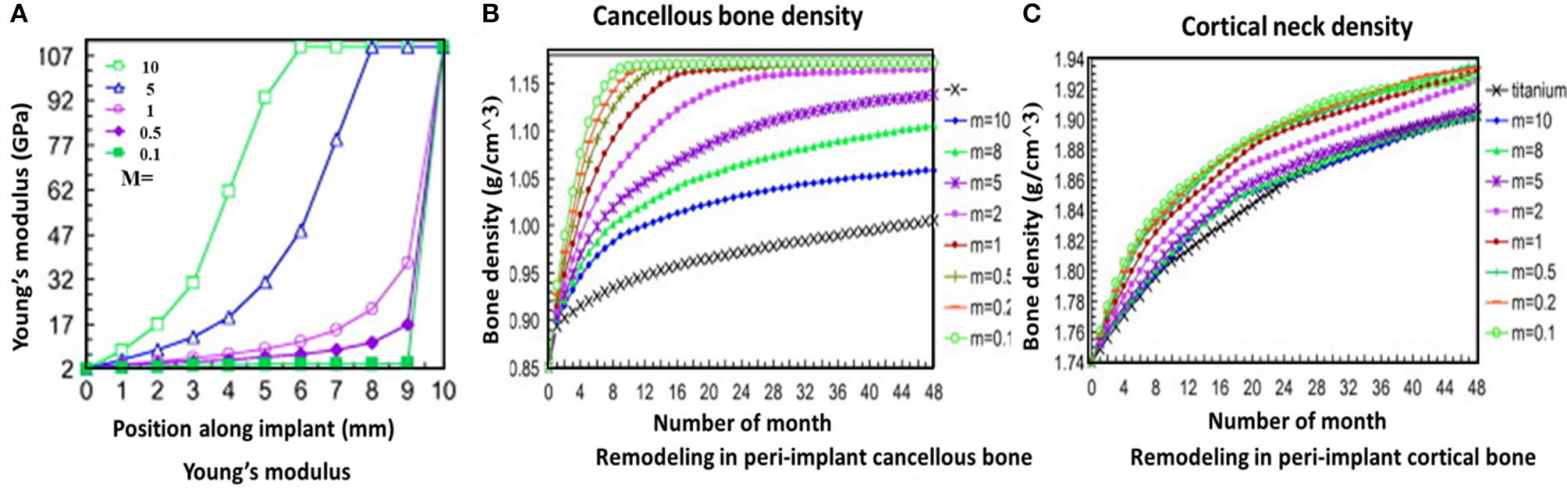
Variation of FGM properties in accordance with FGM characteristics: **(A)** Young's modulus, **(B)** peri-implant bone remodeling against cancellous bone density, and **(C)** peri-implant bone remodeling against cortical neck density with permission from Lin et al. (2009b).

The primary idea behind the utilization of FGMs in dental implants is that the characteristics of the implant can be accurately designed and adjusted to ensure the complete mimicking of the periphery bone tissue and provide the biomechanical necessities according to a specific region of the host bone. Hence, the utilization of FGM dental implants is very beneficial and can enhance integration and implant stability (Lin et al., 2009a). The main advantages of using FGM parts in dental applications are reducing the stress-shielding effect (Hedia, 2005), improving biocompatibility (Watari et al., 2004), inhibition of thermal-mechanical failure (Wang et al., 2007), and providing biomechanical requirements (Yang and Xiang, 2007). Moreover, these modern FGM implants can help to solve the mechanical properties' mismatch issues between implants and native biomaterials, and this is an important problem because it can reduce osseointegration and bone remodeling. In FGM dental implant applications, usually a cylindrical shape is utilized in which the composition varies in the axial direction (Mehrali et al., 2013). The FGM dental implants are designed with varied properties in a certain pattern to match the biomechanical characteristics in a specified region (Lin et al., 2009b). These FGMs are usually composed of collagen HA and Ti as it is known that collagen HA is a key constituent of human bones and other related tissues, and this material can enhance the biocompatibility (Watari et al., 2004). Lin et al. (2009b) studied the effect of FGM design on bone remodeling in a computational remodeling scheme [finite element modeling (FEM)]. In this study, 8 FE models were used, seven of them with varying m values (m = 10, 8, 5, 2, 1, 0.5, and 0.1) plus a model of full Ti for comparison. The m value indicates the ratio in the volumetric fraction of the Ti to HAP/Col compositions, m = 10 shows the richest content of Ti, and m = 0.1 indicates the highest ratio of collagen HAP. Figure 9 shows the results of this investigation. It clearly indicates that reducing the FGM gradient leads to better bone remodeling performance, but unfortunately, the low m values at the same time can reduce the stiffness of implantation. It is suggested that this problem can be solved by a multi-objective optimization scheme.

**Figure 10:**
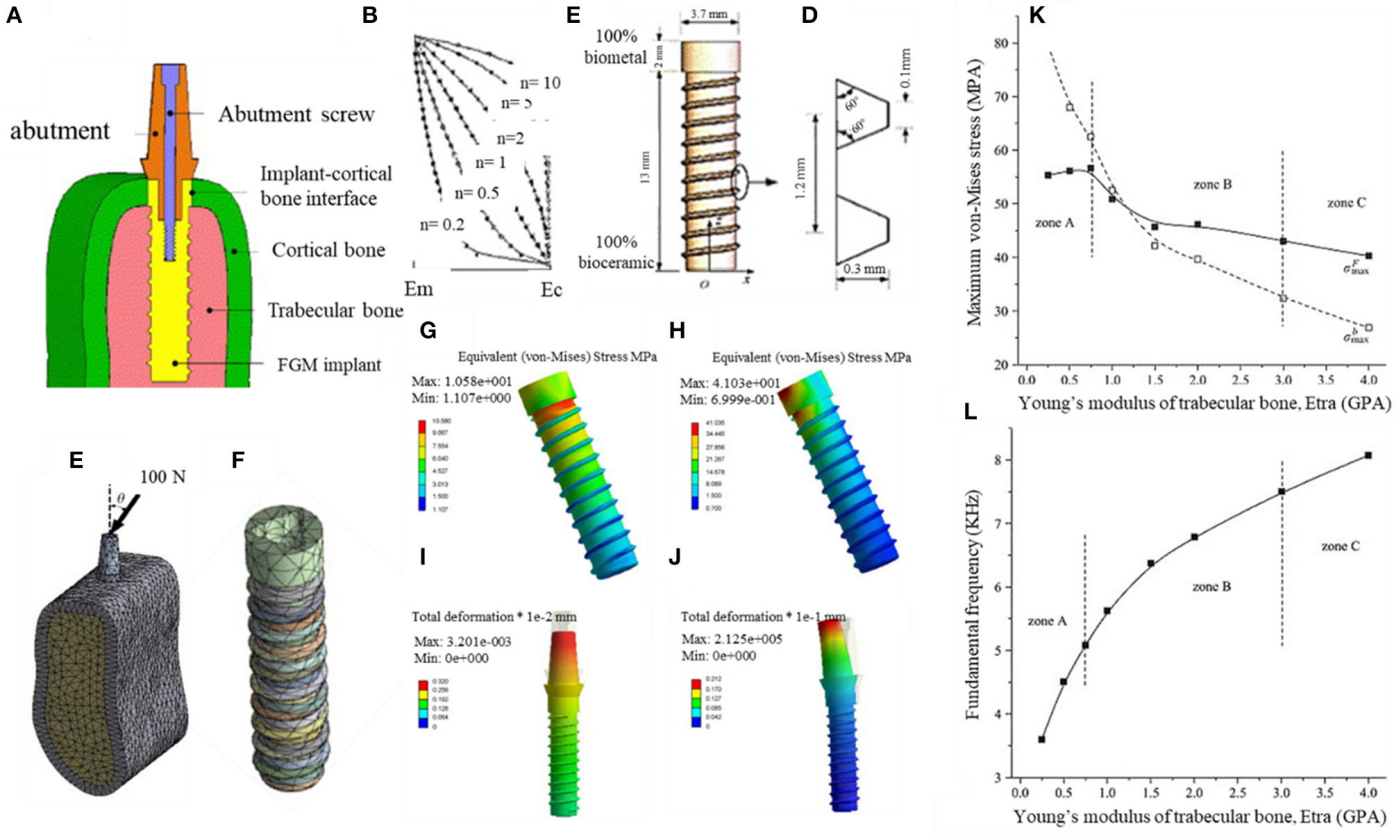
The biomechanical response of three-dimensional FEM designed FGM dental implant in the surrounding bone with permission from Yang and Xiang (2007): **(A)** FGM dental implant to bone condition, **(B)** longitudinal fluctuations in material properties of FGM implant, **(C)** FGM configuration, **(D)** the thread details, **(E)** the implant-bone system, **(F)** FGM implant; the von-Mises stress and displacement distributions in FGM under various conditions: **(G)** vertical occlusal force, **(H)** inclined occlusal force, **(I)** vertical occlusal force, **(J)** inclined occlusal force. Influence of elastic modulus of the peri-implant bones **(K)** on maximum Von-Mises stresses and **(L)** on the FGM fundamental frequency.

Yang and Xiang (2007) investigate a biomechanical response of a dental FGM implant under static and harmonic occlusal forces by the 3-D FEM concept. The implant material was a combination of a bioceramic and a biometal with a smooth composition and properties gradient in the longitudinal direction. In this study, the interaction of the implant and the periphery bone tissues was studied. The samples were produced by the dry method and electric furnace heating (similar to Watari et al., 1998), and varied ratios of Ti to HAP were used (composition varying between pure Ti to 100% HAP) in the longitudinal direction. The dental implant, along with the supporting bone system, is shown in Figure 10A, and other properties of the implant can be seen in Figures 10B–F. The occlusal forces are directly applied to the upper part of the abutment, and then it is directed down to the implant by the screw connection. These occlusal forces mostly are supported by the cortical bone; the large volume percentage of Ti in the upper region of the implant and higher HAP amount in the lower region is favorable because it maintains a satisfactory load-bearing capacity, and also, it can effectively reduce the material mismatch between the implant and the surrounding bone tissues. Figures 10G–J illustrates the von-Mises stress and displacement distributions in FGM under various conditions and confirms that produced stresses are much lower in the middle and lower regions of the implant. Moreover, in Figure 10K, the maximum Von-Mises stress values at various osseointegration phases (zones A = initial, B = mid, and C = complete osseointegration) are compared, and the maximum stresses decrease by increasing the Young's modulus, and the osseointegration condition improves. The condition of the surrounding bone system largely affects the natural frequencies, the variations of frequencies upon the elastic modulus can be used to analyze dental implant performance and its osseointegration. Figure 10L shows that the fundamental frequency considerably enhanced in improved osseointegration conditions. Overall, this study suggests that the utilization of the FGM scheme is a very beneficial procedure for improving the biomechanical response of dental implants (Yang and Xiang, 2007).

The FGM surface coatings can also be used to improve dental implant function (He and Swain, 2009; He et al., 2014), Aldousari et al. (2018) compare three various dental implant models, including (1) a homogenous dental implant with a homogenous coating, (2) a homogenous dental implant with an FGM coating, and (3) a functionally graded implant with a homogenous coating material. It is seen that the third model with the FGM structure and homogenous coating is the most suitable to reduce the stress-shielding effect. Also, the porous FGMs can be used in orthodontic or maxillofacial implants (Becker and Bolton, 1997; Suk et al., 2003). In this regard, Becker et al. (1995) studied various porous biomedical alloys, including 316L stainless-steel, Co-29Cr-6Mo alloy, and Ti-6Al-4V alloy, and confirmed their possible use. Subsequently, Oh et al. (2007) investigated the effect of various pore size in both *in vitro* and *in vivo* biological experiments and showed that the optimum pore size range for fibroblast ingrowth is 5–15 μm, for chondrocyte ingrowth is about 70–120 μm, and for bone regeneration is in the range of 100–400 μm. There are lots of investigations about the porous FGMs. Matsuno et al. (1998) studied the laminated HA/Zr composite with the gradient composition, and its promising properties, such as osteoconductivity and high mechanical strength, show its potential for use in dental and orthopedic implants. Another successful porous FGM Ti dental implant was produced by Traini et al. (2008) *via* direct laser metal sintering with satisfactorily elastic properties, minimum stress shielding effects, and improved long-term performance. Additionally, there are lots of finite element modeling about FGM dental implants showing the effectiveness of the FGM concept in reduction of stresses in periphery tissues (Sadollah and Bahreininejad, 2011; Ichim et al., 2016).

### FGM Orthopedic Implants

Bone tissue is a popular natural FGM structure; hence, it seems very rational to use FGM parts in the treatment of various bone-related issues, such as orthopedic implants. In this regard, biomimetic FGM designs seems to be a prospective solution for implant applications (Boughton et al., 2006, 2013). One example is the BioFI™ arthroplasty design, which emulates bone characteristics of vertebral bone; it successfully mimics the connective intervertebral disk function (Boughton et al., 2010).

**Figure 11:**
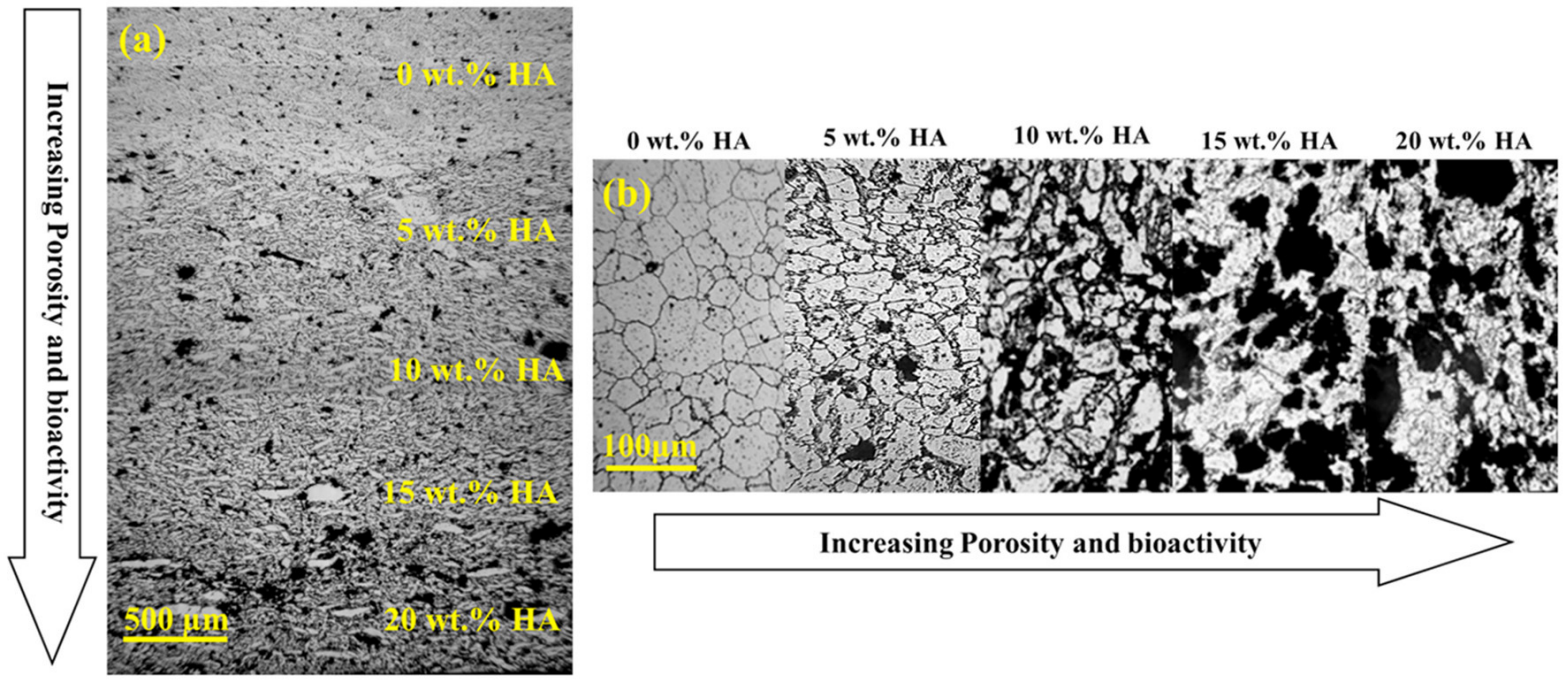
**(a)** A montage micrograph without etching condition of mix-FGMs and **(b)** Montage image after etching with permission from Akmal et al. (2016).

AM technologies have a great potential to fabricate various orthopedic implants from lattice structures and FGMs (Mahmoud and Elbestawi, 2017). The FGM parts can be designed in order to exactly mimic the features of the desired region and maintain the necessities, such as inhibition of the stress-shielding effect and preventing the harmful shear stresses that may be produced in the bone-implant interface regions. There are numerous studies about FGM orthopedic implants, but this review briefly discusses metallic orthopedic FGM implants. Batin and Popa (2011) fabricates a Ti/HA-based implant by powder metallurgy with compositional changes in various layers. It is concluded that, by increasing the HA content, the elastic modulus and compressive strength show the decreasing trend. Akmal et al. (2016) investigate the bioactivity and electrochemical properties of various FGMs produced from stainless steel 316L (SS) reinforced with HA by the powder metallurgy technique. The HA concentration was varied between 0 and 20 wt.% with 5 wt.% increments in each layer; a montage micrograph of successive layers of the mix-FGM in the unetched condition is shown in Figure 11a, and it can be seen that HA has a homogenous distribution in various layers of mix-FGM. Also, an increasing trend of HA from the top to bottom layers is clear, and there is not any crack formation inside the layers or even at the interfaces. To further analyze the interlocking between the matrix and the reinforcement phases, the cross-section of mix-FGM was etched, and its montage micrograph is shown in Figure 11b. The grayish phase demonstrates the SS matrix, and the dull phases are the discontinuous HA phase. Moreover, the microstructure shows the existence of pores, and the porosity has an increasing manner, and grain size reduces in HA content increments. This means that HA content can hinder grain growth by the pinning effect. The electrochemical observations confirm the great performance of these FGMs against corrosion attack in 0.9% NaCl solution.

**Figure 12:**
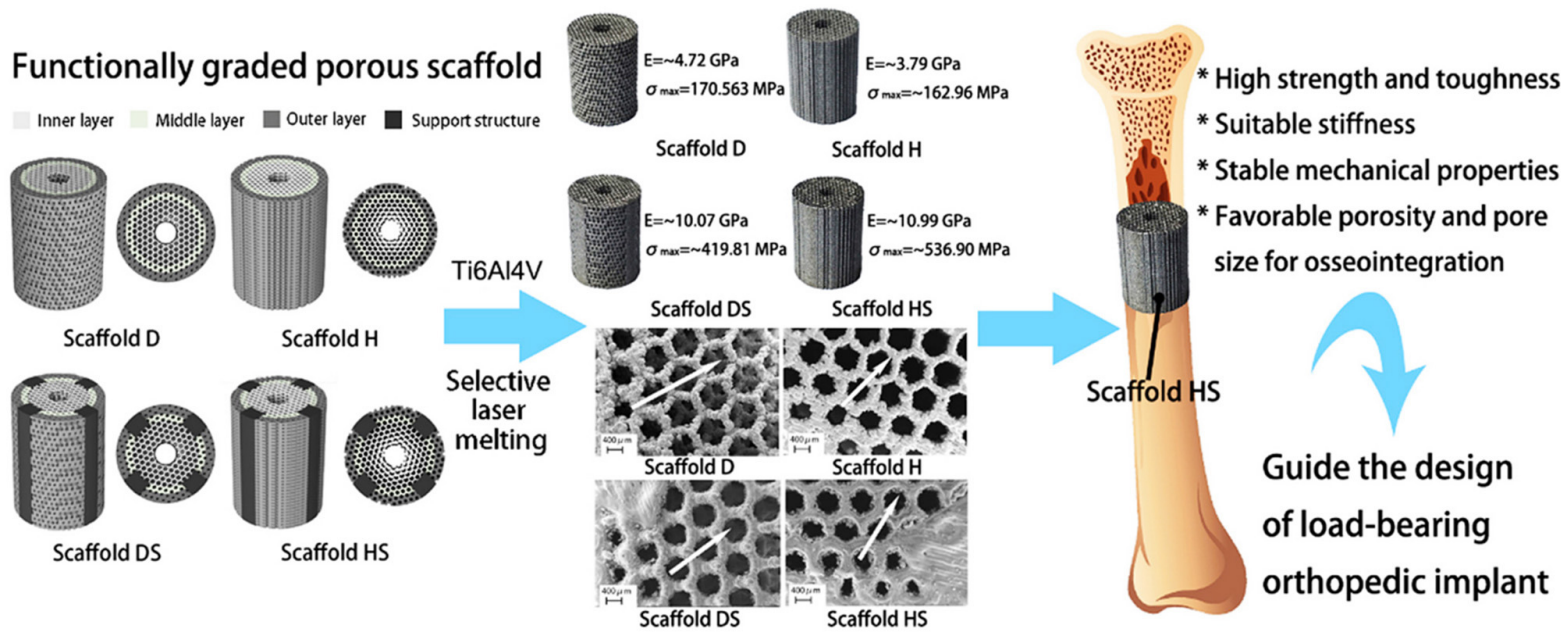
AM manufactured and rationally designed porous Ti6Al4V FGM scaffolds for orthopedic implant applications with permission from Xiong et al. (2020).

As discussed earlier, AM manufacturing has a great impact on the biomedical implant industry, and this is also true of orthopedic implants. Lots of new concepts have been introduced. Xiong et al. (2020) investigate the mechanical properties of SLM-produced Ti6Al4V FGMs with orthopedic implant applications. The rational design in which a radial gradient porous architecture with the potential to mimic the gradient structure of bone is proposed and can be seen in Figure 12. The SLM-produced samples have a linear varying porosity along the radial direction with two porous regions. A high-porosity region is similar to cancellous bone in the inside part for maintaining favorable regeneration and growth of cells and a low-porosity region is similar to cortical bone in the outer areas of the implant with a high load-bearing potential. This design leads to mechanical properties (Young's modulus and yield strength) in the range of natural human bone. It is confirmed that the addition of structural support can substantially improve its compressive strength and toughness along with the preservation of the appropriate elastic modulus. It can also improve the stability of the scaffold and maintain solid energy absorption ability (Xiong et al., 2020). Overall, AM methods are among the best candidates for FGM production in the biomedical field.

The mechanical property gradation (Young's modulus and hardness) was developed by Jung et al. (2009) *via* local heating of a groove rolled (Ti-35%Nb)-4%Sn rod for artificial hip joint application with high strength value at one side and low Young's modulus at the other side; this design can be used for novel orthopedic implants. Hedia et al. (2014) design a cemented stem scheme through an FGM concept, and the result is that the most optimized approach is to use a cemented stem with gradation of Ti and collagen in which the upper stem layer is from Ti and the lower stem layer is composed of collagen. This novel cemented stem design has the potential to eliminate the stress-shielding issue, especially at the nearby medial femoral region.

## Metallic FGMs for Biomedical Applications

**Figure 13:**
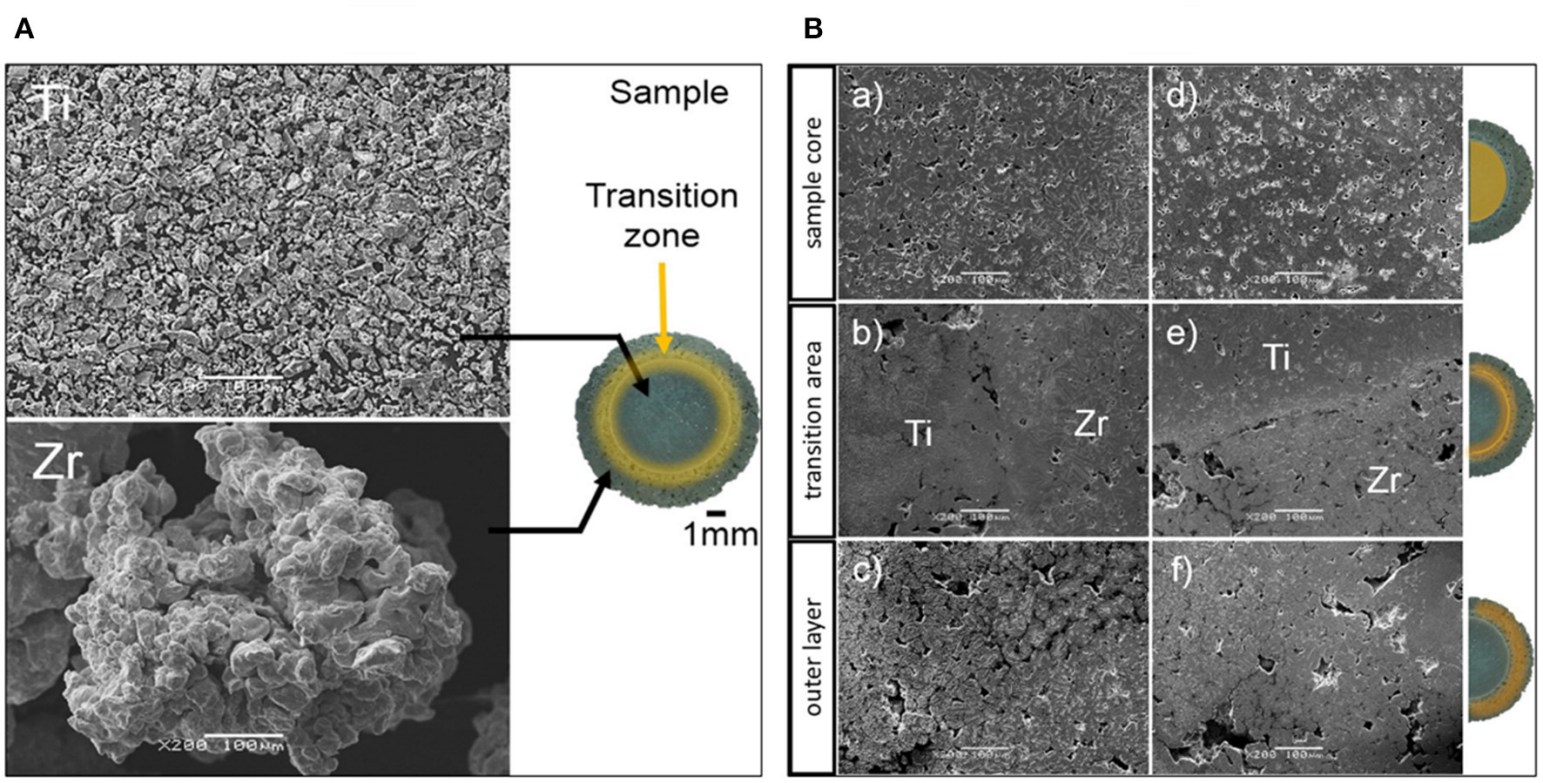
**(A)** The SEM micrographs of initial powders and photo of the sample, including various zones across the sample; **(B)** the SEM micrographs of various zones: (a–c) Ti/Zr(–) produced under the pressure of 500 MPa; and (d–f) Ti/Zr(+) that was produced under 1,000 MPa pressure, reproduced with permission of (Matuła et al., 2020).

In previous sections, the importance and many applications of FGM Ti alloys are discussed, especially the AM manufactured ones. This section aims to analyze other types of FGM manufacturing methods in various metallic and multi-metallic systems. Metal and metal–ceramic composites are one of the most important classes of metallic FGMs, and most of them are designed especially for biomedical applications (Mortensen and Suresh, 1995; Suresh and Mortensen, 1997; Petit et al., 2018). In one study (Matuła et al., 2020), gradient porosities were produced by the powder metallurgy method on titanium/zirconium samples for biomedical applications, and this is shown in Figure 13. It is observed that the sintering process led to fabrication of a non-stoichiometric Zr (Ti) phase due to diffusion along the transition area. Also, gradual microstructural changes are seen in the transition zones, which lead to improved microhardness values. Moreover, the Rietveld refinement results confirm that pressure application during cold isostatic pressing had no significant influence on the unit cells.

**Figure 14:**
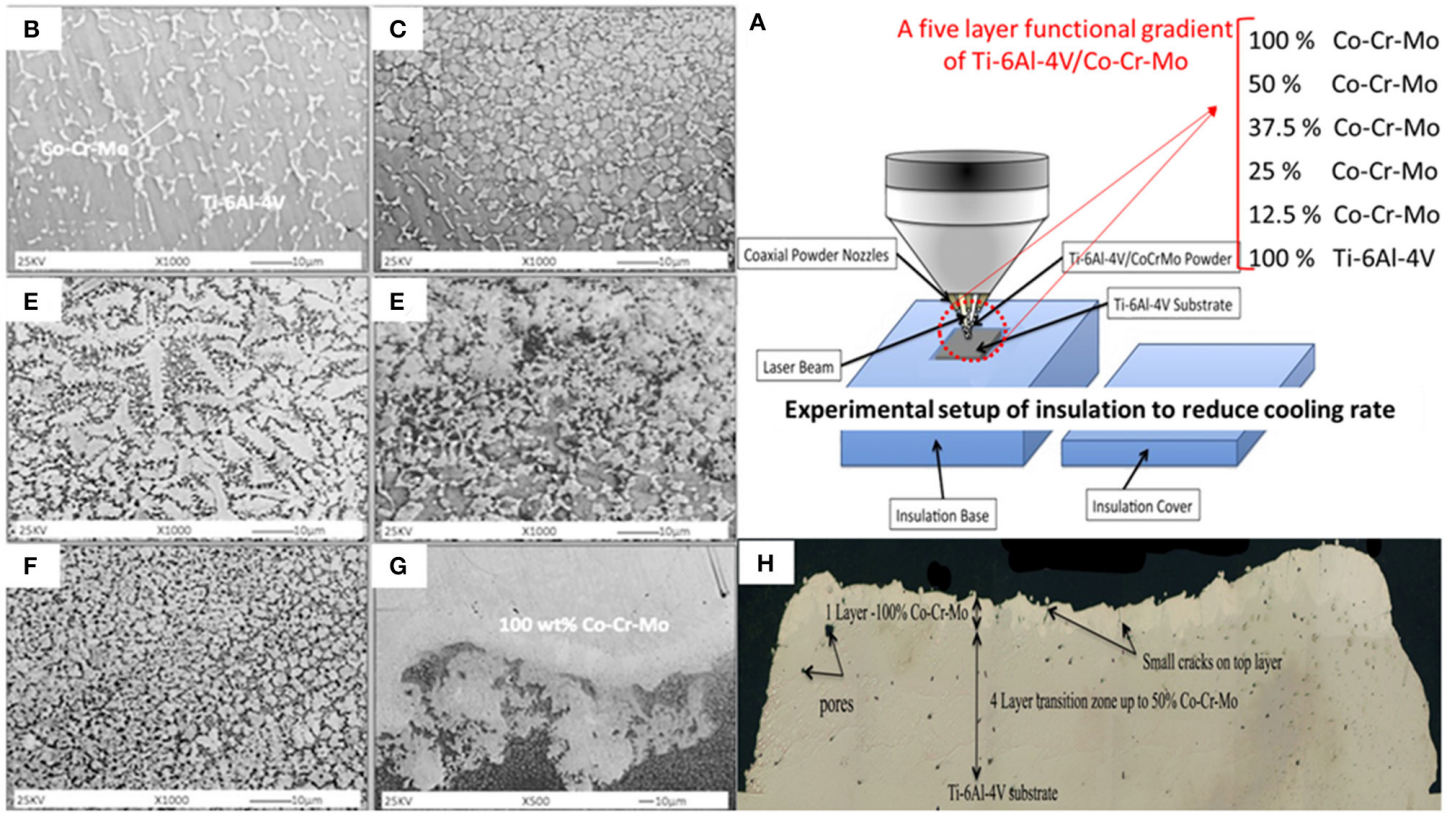
**(A)** The experimental setup of laser deposition, including the insulation cover and the composition of five-layer functional gradient of Ti-6Al-4V/Co-Cr-Mo samples; **(B–G)** Corresponding SEM images of gradient Ti-6Al-4V/Co-Cr-Mo composite structure 1000×, **(B)** 10 wt.%, **(C)** ~20 wt.%, **(D)** ~30 wt.%, **(E)** ~35 wt.%, and **(F)** ~40 wt.% Co-Cr-Mo material, **(G)** the interface zone between 50/50 wt.% Ti-6Al-4V/Co-Cr-Mo and 100 wt.% Co-Cr-Mo; **(H)** Cross-section of the sample showing the Co-Cr-Mo gradient coating and some crack and pore formation on Ti-6Al-4V with up to 100% coating on the top layer; images were reproduced from Wilson et al. (2013) with permission.

Wilson et al. (2013) fabricate a functionally gradient bio-coating containing Co-Cr-Mo material (0–100%) on Ti-6Al-4V substrate *via* laser deposition (Figure 14A). Also, the SEM images of gradient Ti-6Al-4V/Co-Cr-Mo composite structure is shown in Figures 14B–G. This FGM material is aimed at reducing the influence of thermal expansion differences between two biomaterials through insolation cover. The results show the successful formation of a composite with excellent microscopic integrity without gross crack formation (Figure 14H) and favorable bonding strength between Co-Cr-Mo-alloy coatings and Ti-6Al-4V substrates. The gradual microhardness improvement by more than 83% was seen from Ti-6Al-4V substrate to the 50:50 composition layer. The average bonding strength was about 63.4 MPa; it was seen that the particles of Co-Cr-Mo coating were removed from the Ti-6Al-4V substrate through the adhesive mechanism. Also, the tensile strength was about 34.5 MPa and was much greater than the required minimum coating strength according to the ASTM standards. Overall, functionally gradient Ti-6Al-4V/Co-Cr-Mo material has a great potential to reduce the effects of thermal expansion differences between two biomaterials and, according to favorable microhardness, tensile, bonding strength, and biocompatibility, can be considered as a good FGM candidate for biomedical applications.

**Figure 15:**
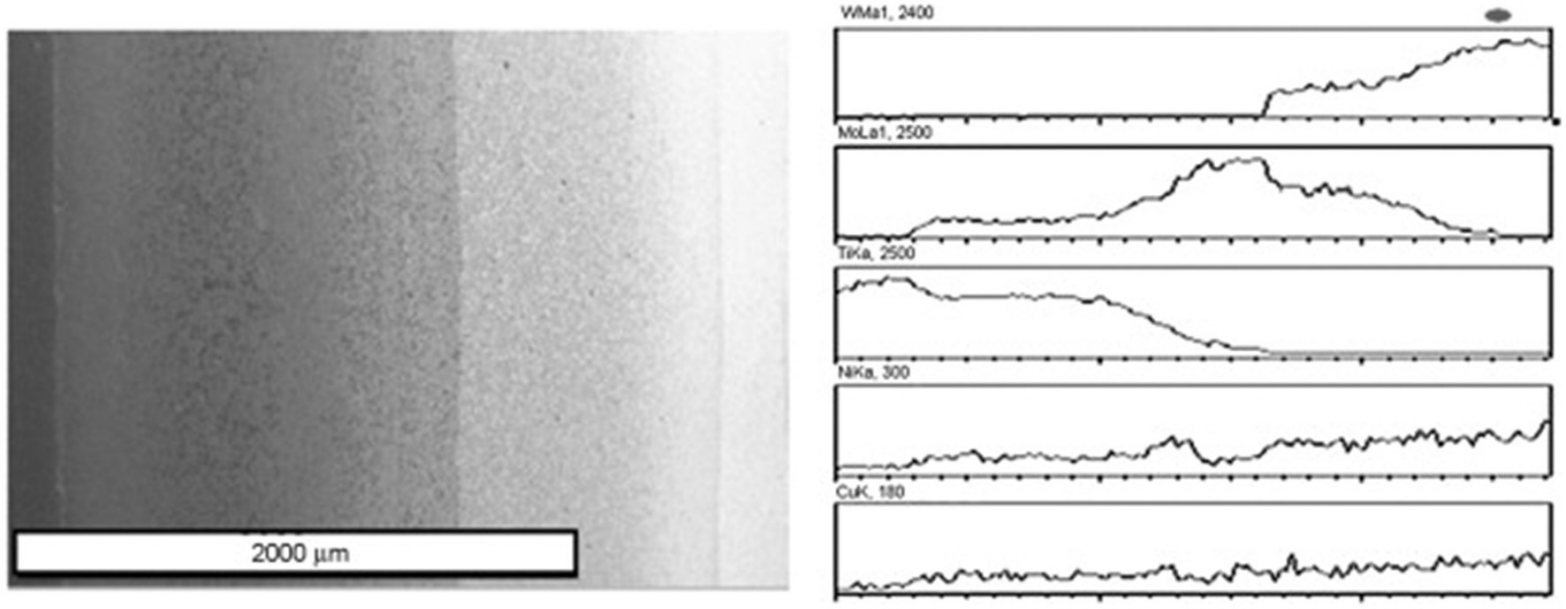
The electron micrograph and linear distributions of elements along the cross-section of W–Mo–Ti FGM with permission from Yu et al. (2003).

One of the approaches to produce various metallic FGMs is sedimentation and flotation because gravity is a phenomenon that is active everywhere on the earth. By exploiting this method, particles with varied size, density, and mass can move differently in liquid metals and alloys, leading to formation of graded structures, and this can be done by thermal controlling of die cooling (Drenchev et al., 2002). It is believed that sedimentation and flotation is one of the best available concepts to fabricate large-scale FGMs with very smooth and gradual variations in composition and microstructure. Yu et al. (2003) produce a sort of W–Mo–Ti FGM with density gradients *via* a co-sedimentation method, and the samples were deposited after layer-by-layer settlement of the corresponding powders. First, the pure Ti layer is settled; second, the Ti–Mo graded layer; third, the Mo–W graded layer, and finally, the pure W layer has been settled. Also, a minor amount of Ni and Cu powders were used as sintering activators. After powder treatment, a set of suspensions was prepared and poured successively into a sedimentation container. After attaining full particle sedimentation, the deposit body compacted and sintered at 1,200°C under a pressure of 30 MPa in a vacuum furnace; Figure 15 shows the electron micrograph and linear distributions of elements along the cross-section of W–Mo–Ti FGM.

**Figure 16:**
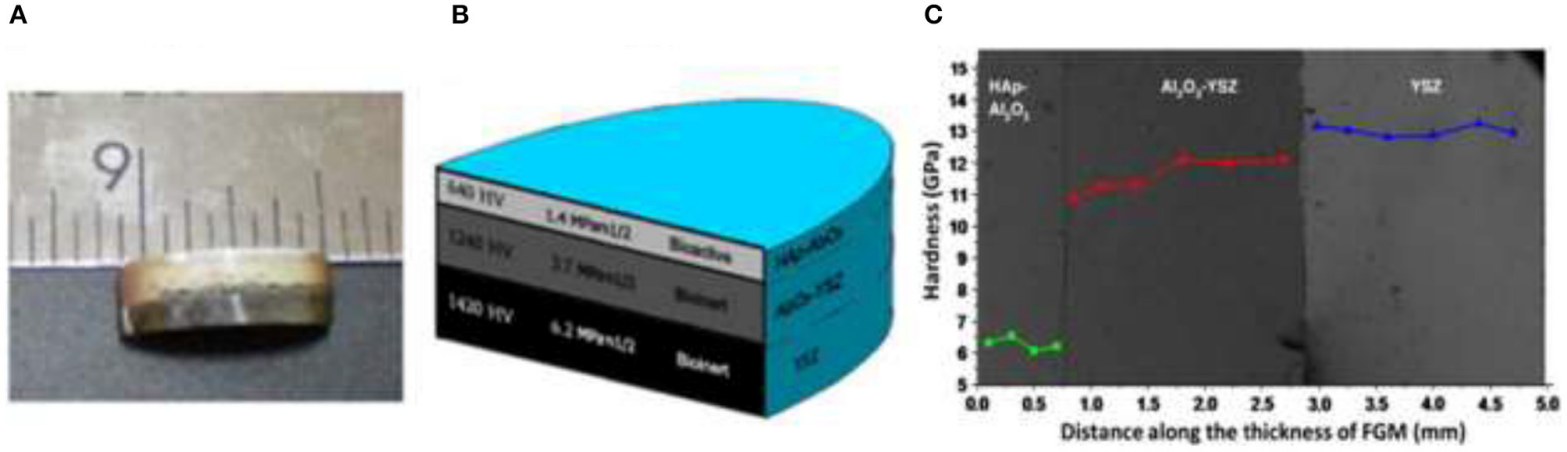
**(A)** The spark plasma sintering (SPS) processed FGM sample; **(B)** schematic representation of FGM structure, including hardness, toughness, and biological response of different layers; **(C)** hardness values across the FGM cross-section with permission from Afzal et al. (2012).

The structural, physical, and mechanical properties of stainless steel (SS-316L)/HA and SS-316L/calcium silicate (CS) FGMs that were produced by powder metallurgical solid-state sintering were studied by Ataollahi et al. (2015). It is shown that high-temperature sintering led to the reaction between compounds of the SS-316L and HA although it has no considerable effect in the SS-316L/CS composite. Uniaxial compressive mechanical experiments show sharp reduction in SS-316L/HA with increasing HA content up to 20 wt.% and gradual variations in SS-316L/CS composites with CS content up to 50 wt.%. Also, the mechanical properties of SS-316L/HA FGM decreased with temperature increment although it showed improvement for the case of SS-316L/CS FGM. It is concluded that the SS-316L/CS composites and their FGMs have much better compressive mechanical properties compared to the SS-316L/HA composites and their FGMs. Moreover, the SS-316L/CS FGMs with better mechanical and enhanced gradation in physical and structural properties are among the suitable candidates for potential use in the load-bearing application (Ataollahi et al., 2015). A novel FGM by introduction of aluminum oxide and an yttria stabilized zirconia (YSZ) cushion layer was produced *via* the SPS process for potential bone implant applications (Afzal et al., 2012). The main goal of this FGM design was to attain a smooth gradation of functionality, including improved toughness of the bulk, and retained biocompatibility of the surface. In this regard, HA and YSZ with, respectively, ~1.5 and ~6.2 MPa.m^1/2^ fracture toughness were attached to a transition layer of Al_2_O_3_, inducing the minimum gradient of mechanical properties with fracture toughness of ~3.5 MPa.m^1/2^ (Figure 16). Measurement of hardness, fracture toughness, and cellular activities across the FGM cross-section illustrates the successful achievement to smooth transition in the HAp-Al_2_O_3_-YSZ FGM composite. Also, L929 fibroblast and Saos-2 osteoblast cell culturing showed the promising cell proliferation and adhesion on the FGM surface. It seems that this HAp-Al_2_O_3_-YSZ FGM has favorable properties for its possible utilization in bone implants.

**Figure 17:**
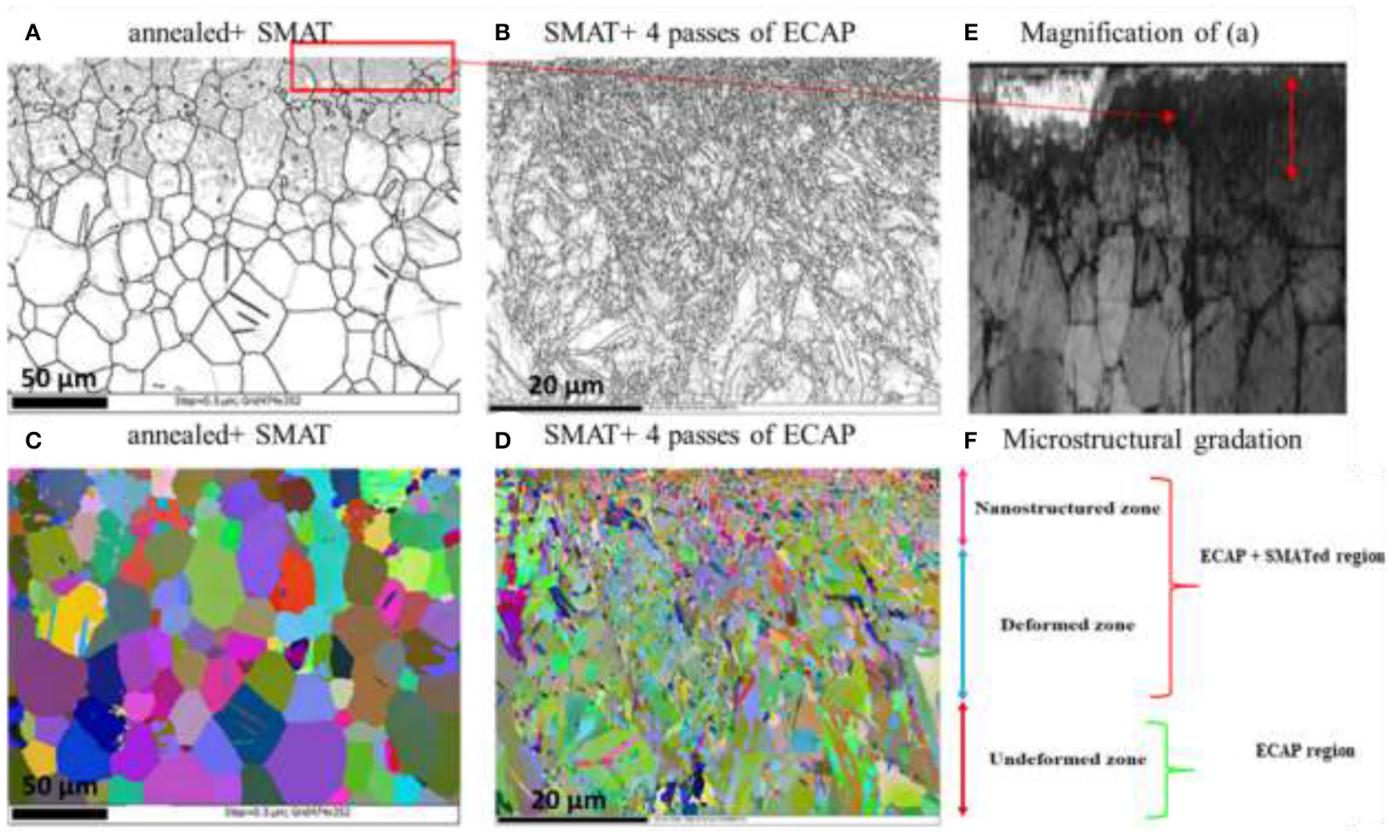
The EBSD micrographs of Ti FGM with grain size gradation in cross-section; **(A,C)** the annealed+ SMATed sample; **(B,D)** SMAT+ 4 passes ECAPed sample (black and white images **(A,B,E)** are grain boundary maps, and colored images **(C,D)** are Euler maps; **(E)** the magnified image of **(A)**; **(F)** shows the microstructural gradation in a cross-section of the SMAT+ECAP sample, reproduced with permission from Attarilar et al. (2019b).

Another interesting study (Attarilar et al., 2019b), uses the grain size variation concept to fabricate pure Ti FGM material for biomedical applications with enhanced mechanical and biocompatibility properties. For this aim, a combined severe plastic deformation method was used. First, an ultrafine-grained (UFG) structure in the bulk of a material was produced by equal channel angular pressing (ECAP) in order to strengthen the bulk. Subsequently, the surface of the material reached a nanosized (NS) microstructure *via* surface mechanical attrition treatment (SMAT) (Figure 17). The surface nanostructures were considerably beneficial because they enhanced roughness, wettability, and TiO_2_ oxide formation and led to better biomechanical bonding of cells and, finally, improved biological response. Moreover, nanoindentation experiments showed the gradual microhardness improvement values from the UFG substrate to the NS surface layer, and the osteosarcoma G292 cell culturing confirmed the improved biological response through cell viability, alkaline phosphatase (ALP) activity, and cell attachment experiments (Attarilar et al., 2019b). A very interesting aspect of this research is that the successful FGM structure was achieved without the need to create composites and additional materials, so the bonding and separation issues encountered between different layers was completely mitigated.

**Figure 18:**
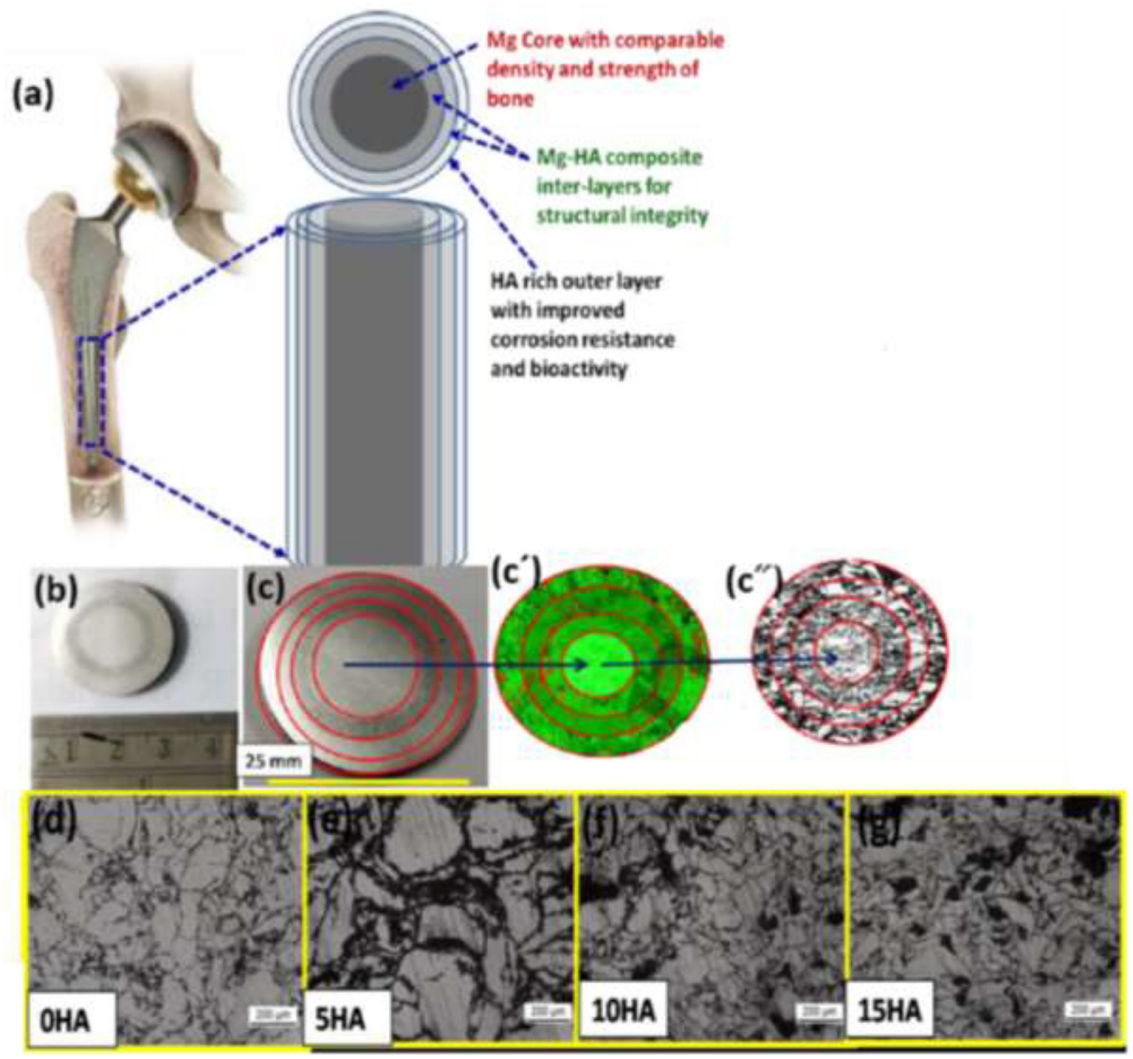
**(a)** The schematic of Mg-based co-centric FGM composite, **(b,c)** image of FGM sample with different co-centric layers, (c′,c″) the corresponding EDS and optical morphology images in various FGM layers, **(d–g)** optical micrographs of individual layers with various HA constituent with permission from Dubey et al. (2020).

A magnesium-based FGM composite for temporary orthopedic implant applications was produced by the SPS method due to its exceptional biodegradation behavior and mechanical characteristics similar to natural bone, which reduces the unfavorable stress-shielding effect (Dubey et al., 2020). The major drawback of Mg is its high *in vivo* corrosion rate, so the Mg-HA FGM production aimed to overcome this limitation. The FGM consisted of Mg at the core area with gradual increments of HA toward the outer layers (Figure 18). It is confirmed that this FGM has the potential to successfully attain a high corrosion-resistant property (about 154% improvement) in the surface along with uniform mechanical integrity distribution across the FGM structure. Moreover, it improved biocompatibility, osteoconductivity, and excellent osteogenic differentiation confirmed by MG63 cell culturing (Dubey et al., 2020). It seems that utilization of Mg-based FGM is a promising candidate with favorable properties for temporary orthopedic implant applications.

### Gradient Nanostructured (GNS) Metals and Alloys

One of the important classes of FGMs is called gradient nanostructures (GNS), including gradient nanograined, nano-twinned, and nano-laminated metals and alloys. Sometimes, these GNS materials can exhibit extraordinary mechanical properties, such as strain hardening, strength–ductility synergy, enhanced fracture and fatigue resistance, and significant corrosion and wear resistance, that are not easily found in materials with homogeneous or random structures. Usually GNS metals and alloys are fabricated with a gradation in the microstructure (grain size, twin thickness, and/or lamellar thickness) from the surface to the depth of the sample as illustrated in Figure 2B (Li X. et al., 2020).

**Figure 19:**
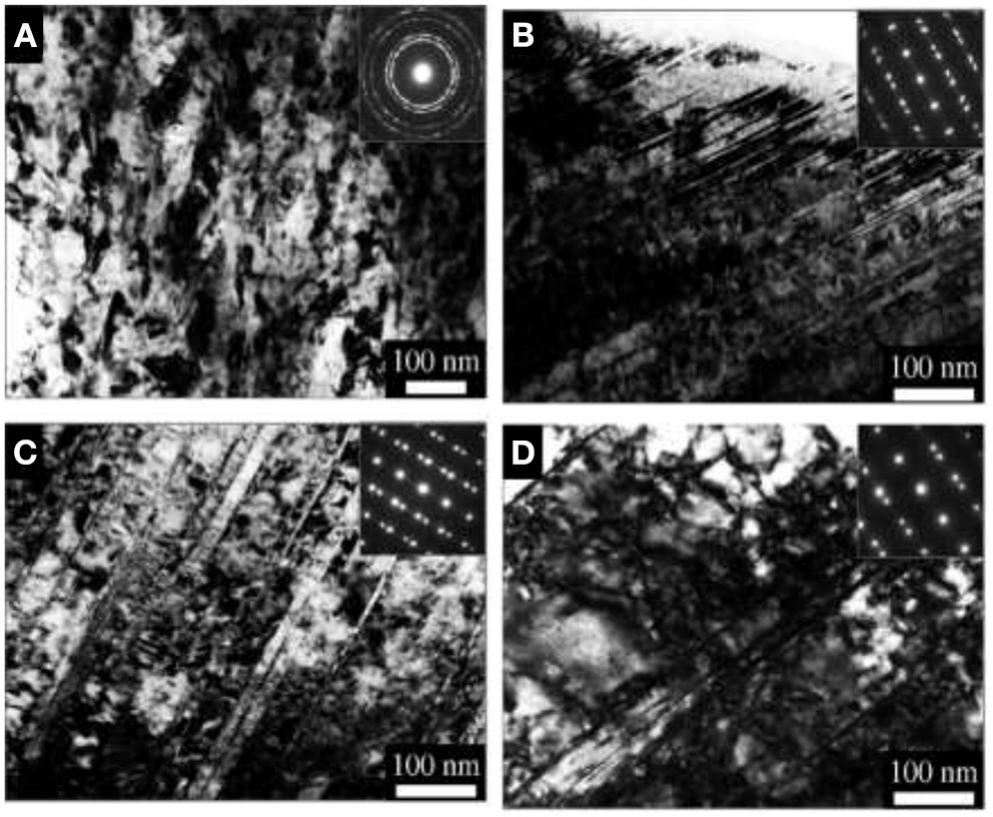
The TEM cross-sectional micrographs of the SMGT Fe–Mn sample at different depths from the top surface. **(A)** ~10 μm; **(B)** 45 μm; **(C)** 188 μm; **(D)** 440 μm, inset pictures are the corresponding selected area electron diffraction patterns with permission from Wang et al. (2013).

One of the good examples of GNS materials is the nano-twin structure. Wang et al. (2013) produced an architectured surface layer with a gradient decrease in twin density in a Fe–Mn austenitic steel *via* the surface mechanical grinding treatment (SMGT) technique. This gradation in twin density corresponds to a gradient hardness reduction from 5.3 GPa in the top layer to about 2.2 GPa in the coarse-grained core. Also, the hardness dependence to twin thickness and superior strength–ductility synergy was observed in these samples; Figure 19 shows the TEM cross-sectional micrographs of the SMGT Fe–Mn sample at different depths from the top surface. In the topmost layer (~10 μm thick, Figure 19A), the existence of nanosized grains with random orientations is clear. In the underneath layer (45 μm), a mixed microstructure of nanosized grains and nanoscale twin bundles are seen. Then, as we get closer to the depths of the sample, it can be seen that, gradually, the twin density and nano-twin bundle volume fraction increases while the volume fraction of nanograins drops.

**Figure 20:**
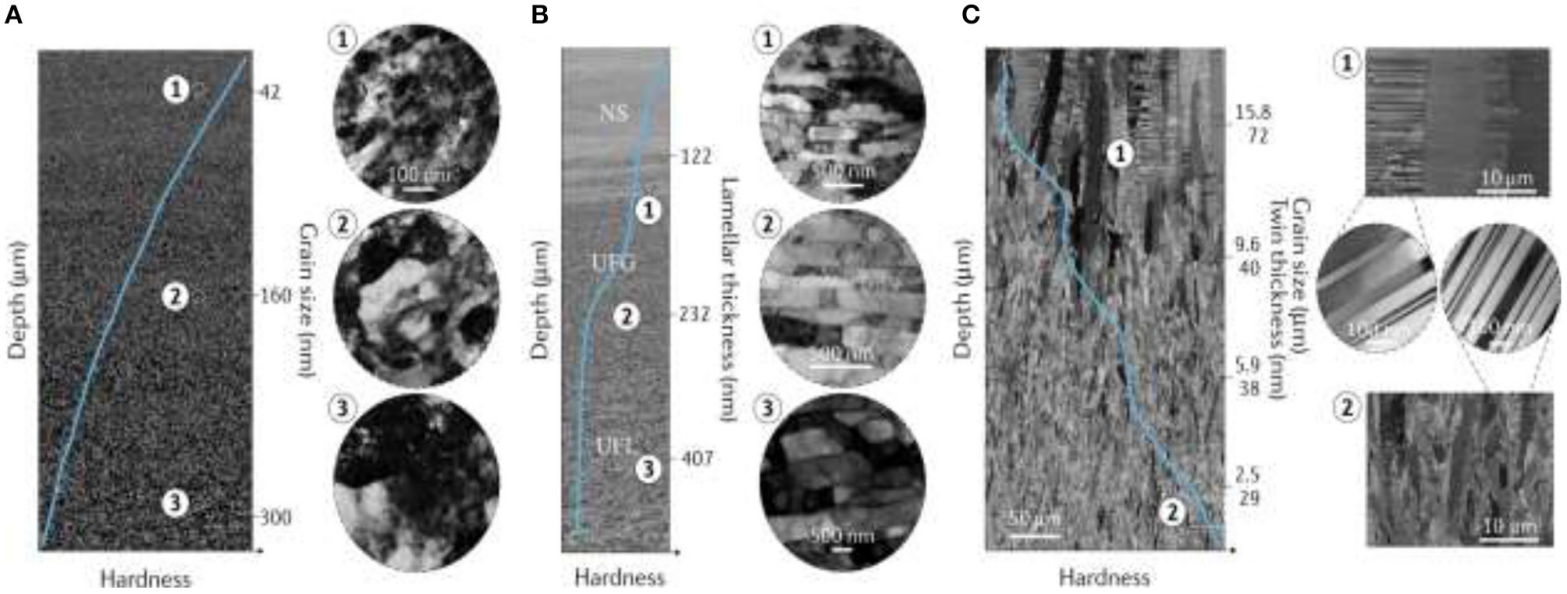
**(A)** The SEM micrograph of gradient nanograined Cu confirming the grain size increase with depth with TEM micrographs from various depths. **(B)** The SEM micrograph showing the microstructure evolution in gradient nano-laminated Ni, including three distinct regions with nanostructures (NS), ultrafine-grained (UFG), and ultrafine-laminated (UFL) structures with depth increase along with TEM pictures from different depths. **(C)** SEM micrographs of the gradient nano-twinned structure in Cu confirming the reduction of grain size and twin thickness with depth along with TEM images of twin and grains at various depths. The solid blue lines inside SEM images present local hardness magnitude with respect to depth in the gradient layer. Image reused with permission from Li X. et al. (2020).

As another example of GNS, the gradient nano-grained (GND) Cu with almost twice the yield stress of conventional Cu samples, significant strain softening, and excellent plasticity, was fabricated by the SMGT process (Chen et al., 2017) (Figure 20A). It was seen that the GND Cu layer could accommodate massive plastic strains. Liu et al. (2015) use the SMGT process to fabricate a nano-laminated structure in nickel by utilization of varied gradation of strain and strain rates in the subsurface layer with about 10–80 μm depth from surface; the existence of 2-D laminated structures with low angle boundaries and strong deformation textures with an average thickness of about 20 nm were observed (Figure 20B). It was confirmed that deformation of these nano-laminated structures happened due to dislocation slip and deformation twining at the nanoscale, finally leading to formation of nano-sized equiaxed grain structure. Another GND Cu structure was fabricated by direct-current electrodeposition with a controllable, homogeneous nano-twinned component (Cheng et al., 2018). It was seen that the structure includes a large number of preferentially oriented nanometer-scale twin boundaries enclosed within the micrometer-scale columnar-shaped grains (Figure 20C).

## The 4-D Printing in Metallic FGMs

Four-dimensional (4D) printing methods have the same principles as 3-D printing and are very similar to it; the main difference is the layer-by-layer formation of smart materials in which one more dimension is added to the material. Therefore, the 4-D printed part can transform its shape with respect to other physical and chemical parameters, such as temperature, pressure, humidity, light, magnetic and electric fields, and even time. These 4-D printed parts have a programmable nature that is a very favorable property; they can easily be controlled and respond according to the environmental condition and design requirements. This technology has many promising applications especially in customized implants and smart biomaterials (Javaid and Haleem, 2020). There are very limited studies about 4-D printing of metallic parts, most of the studies are about the 4-D printing of polymeric materials. Chen et al. (2019) introduces an innovative procedure capable of 3-D printed multimetal *via* an electrochemical 3-D printer system. This modern technology can produce bimetallic structures through the selective deposition of various metals; hence, a temperature-responsive reaction can be programmed into the 4-D printed metallic parts. Although the studies in this area are very rare, due to the favorable and controllable properties of 4-D printing technologies, it seems that they have a very bright future in the production of smart metallic biomaterials.

## Future Scenery

The FGM concept was an evolutionary pathway toward the design of multifunctional systems with lots of advantages, including smart design, potential to mimic natural biological systems, rapid and integrated designs, utilization of CAD systems and FEM-based schemes, enhanced properties, and conquering limitations such as the stress-shielding effect, etc. Although these are valuable advantages, numerous issues should still be solved, such as lack of systematic and detailed guidelines for various applications, design principles, involved mechanisms, widely accepted standards, and new technologies. Hence, future investigations should focus on overcoming these issues and providing comprehensive databases; improving techniques and related computer models, software, etc.; fabrication of modern devices; enhancing the compatibility of these parts with the entire system both biologically and mechanically; improving the fabrication resolution with the capacity to produce slight gradations; finding new methods to carefully tailor the distribution of phases, pores, and other constituents; upgrading the utilized materials; application of new concepts such as 4-D designs (Javaid and Haleem, 2019); bioprinting (Dwivedi and Mehrotra, 2020); metamaterials (Zhu et al., 2019; Sepehri et al., 2020); and so on. In this regard, considering the great impact of AM technology, it should be further developed because it still is not applicable in real industrial conditions.

FGMs can be utilized in various areas, including biomedicine, structural parts, aviation, thermal management, energy-absorbing systems, optoelectronic, electromagnetic interference shielding (EMI), and even geological models that can analyze earthquakes and natural landslide disasters (Li Y. et al., 2020). The FGM concept can also be used in 4-D printing by the functionally graded additive manufacturing (FGAM) concept in which a single AM process incorporates the gradational mixing of materials in order to construct freeform geometries with changing properties within one component (Sudarmadji et al., 2011; Pei et al., 2017). Unfortunately, the existing AM technologies only have prototyping advantages with very limited functionality, so extensive research progress should be devoted to this field. The FGM designs profiting from the 4-D concept (Javaid and Haleem, 2020) can lead to the fabrication of *de novo* and smart parts that can completely change our lives; these intelligent, multifunctional systems can be used in the treatment of various diseases and hazardous tasks for humans. In brief, it can be said that FGMs have a great potential to influence the future of biomedicine and industry through their intricate gradients and multifunctional nature, and they demonstrate great prospects in the future of our technologies. In particular, it would open new horizons in biomedicine.

## Conclusions

FGMs as a modern concept of materials attract much attention from academicians, the medical field, and industry. Because of the graded nature of bone and other natural systems, utilization of FGMs are very beneficial in biomedicine, and they can improve the overall performance of implants both biologically and biomechanically. This review paper comprehensively discusses the design criteria, techniques, applications, pros, and cons of FGMs, especially in biomaterials. In this regard, different FGM production methods are studied, especially additive manufacturing methods that are very capable in the production of complex FGM structures with high resolution and with multifunctional characteristics. Also, other methods, such as powder metallurgy, gas-based, liquid-based, and solid-phase processes, are introduced. The biological concept of FGM design and the functional design of FGMs in biomedical applications are discussed thoroughly. The utilization of FGM design in dental and orthopedic implants is specifically considered in which the FEM-based designs are used to simulate natural tissues. The effect of FEM design in biological and biomechanical performance of implants is the focus of research because the main aim of these graded designs is to affect these properties. Subsequently, the future scenery of FGM, its applications, and the limitations that should be overcome were briefly mentioned. The modern concepts that can assist the more rapid development of FGM and its applications were introduced, including bioprinting, 4-D designs, metamaterials, etc. Finally, it can be said that FGMs, as a modern scheme, can substantially influence the future of biomedicine and its application, but still, it needs to be further developed from both a technological point of view and scientific aspect, and it has a long way to go to reach its optimum condition and vast industrialization. Hence, this review paper aimed to illuminate the way toward the modern, smart, and advantageous FGM biomaterials.

## Author Contributions

HS wrote the main part of the manuscript. PZ and JL contributed to the writing FGMs techniques section, biomedical applications, prepared, and formulated the references. PZ made major contributions particularly in choosing the figures. PZ, JL, CL, and LW made significant contribution to the revision stage. All authors contributed to the article and approved the submitted version.

## Conflict of Interest

The authors declare that the research was conducted in the absence of any commercial or financial relationships that could be construed as a potential conflict of interest.
